# A photorespiratory bypass increases plant growth and seed yield in biofuel crop *Camelinasativa*

**DOI:** 10.1186/s13068-015-0357-1

**Published:** 2015-10-29

**Authors:** Jyoti Dalal, Harry Lopez, Naresh B. Vasani, Zhaohui Hu, Jennifer E. Swift, Roopa Yalamanchili, Mia Dvora, Xiuli Lin, Deyu Xie, Rongda Qu, Heike W. Sederoff

**Affiliations:** Department of Crop Science, North Carolina State University, Campus Box 7287, Raleigh, NC 27695-7287 USA; Department of Plant and Microbial Biology, North Carolina State University, Campus Box 7612, Raleigh, NC 27695-7612 USA

**Keywords:** Camelina, Seed yield, Biofuel, Photorespiratory bypass, Photosynthesis

## Abstract

**Background:**

*Camelina sativa* is an oilseed crop with great potential for biofuel production on marginal land. The seed oil from camelina has been converted to jet fuel and improved fuel efficiency in commercial and military test flights. Hydrogenation-derived renewable diesel from camelina is environmentally superior to that from canola due to lower agricultural inputs, and the seed meal is FDA approved for animal consumption. However, relatively low yield makes its farming less profitable. Our study is aimed at increasing camelina seed yield by reducing carbon loss from photorespiration via a photorespiratory bypass. Genes encoding three enzymes of the *Escherichia coli* glycolate catabolic pathway were introduced: glycolate dehydrogenase (GDH), glyoxylate carboxyligase (GCL) and tartronic semialdehyde reductase (TSR). These enzymes compete for the photorespiratory substrate, glycolate, convert it to glycerate within the chloroplasts, and reduce photorespiration. As a by-product of the reaction, CO_2_ is released in the chloroplast, which increases photosynthesis. Camelina plants were transformed with either partial bypass (GDH), or full bypass (GDH, GCL and TSR) genes. Transgenic plants were evaluated for physiological and metabolic traits.

**Results:**

Expressing the photorespiratory bypass genes in camelina reduced photorespiration and increased photosynthesis in both partial and full bypass expressing lines. Expression of partial bypass increased seed yield by 50–57 %, while expression of full bypass increased seed yield by 57–73 %, with no loss in seed quality. The transgenic plants also showed increased vegetative biomass and faster development; they flowered, set seed and reached seed maturity about 1 week earlier than WT. At the transcriptional level, transgenic plants showed differential expression in categories such as respiration, amino acid biosynthesis and fatty acid metabolism. The increased growth of the bypass transgenics compared to WT was only observed in ambient or low CO_2_ conditions, but not in elevated CO_2_ conditions.

**Conclusions:**

The photorespiratory bypass is an effective approach to increase photosynthetic productivity in camelina. By reducing photorespiratory losses and increasing photosynthetic CO_2_ fixation rates, transgenic plants show dramatic increases in seed yield. Because photorespiration causes losses in productivity of most C3 plants, the bypass approach may have significant impact on increasing agricultural productivity for C3 crops.

**Electronic supplementary material:**

The online version of this article (doi:10.1186/s13068-015-0357-1) contains supplementary material, which is available to authorized users.

## Background

*Camelina sativa* is an oilseed crop belonging to the Brassicaceae family. The plant is an attractive crop for sustainable biofuel production on marginal lands [1, 2]. Its seeds contain about 40 % oil by weight (twice that of soybean seeds) [3, 4], which can be readily converted to industrial lubricants, biodiesel [5, 6] and jet fuel [7, 8]. The camelina seed meal is FDA approved for animal consumption. However, camelina plants have a relatively low seed yield [9], and are therefore currently not profitable enough in cultivation [10]. Increasing seed yield of any oil crop will reduce the competition for land with food and feed production and conservation. We have addressed this issue by increasing yield through reducing photorespiratory carbon and energy losses.

**Fig. 1:**
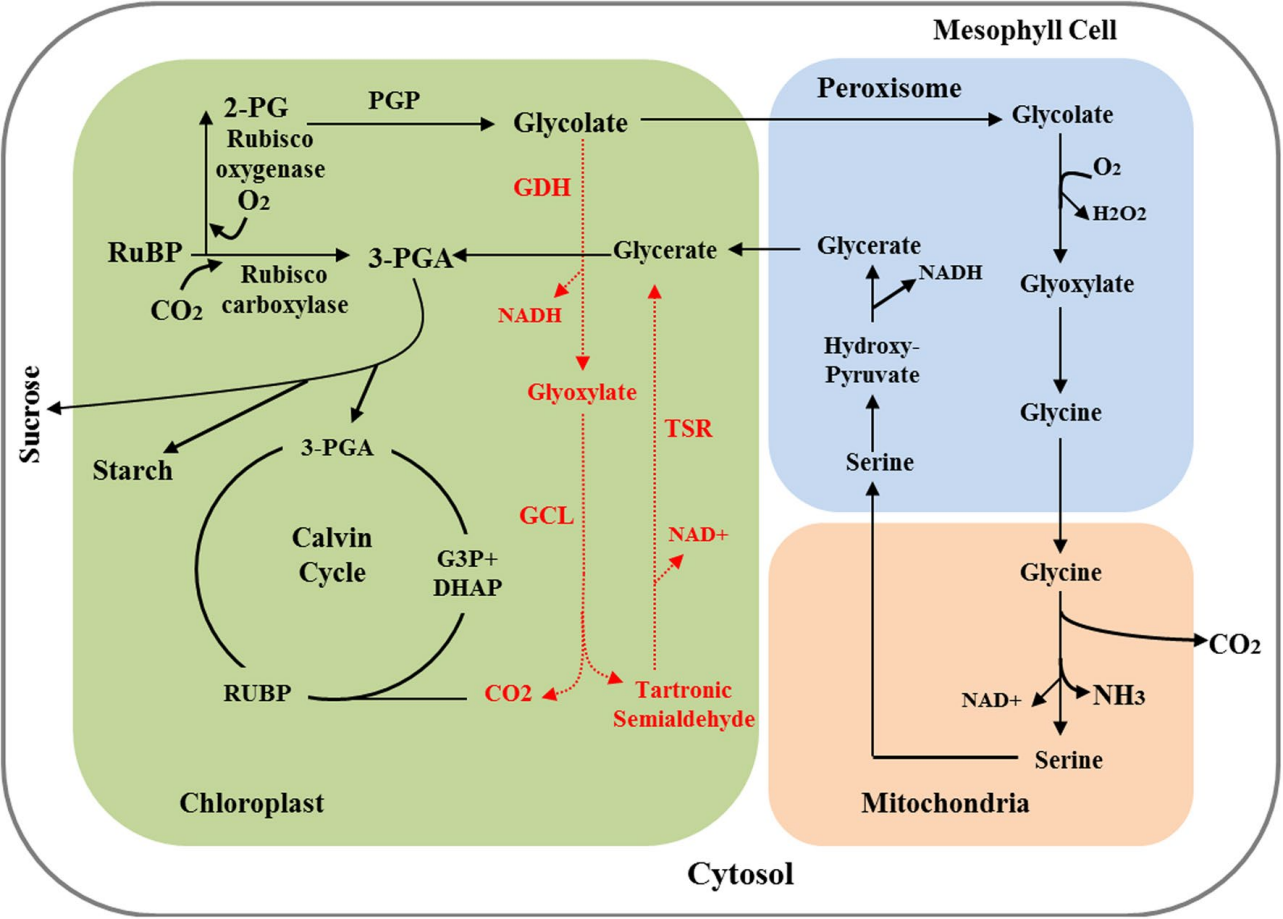
Overview of the photorespiratory bypass. *RuBisCO* ribulose-1,5-bisphosphate carboxylase/oxygenase, *RuBP* ribulose-1,5-bisphosphate, *PGP* phosphoglycolate phosphatase, *GDH* glycolate dehydrogenase, *GCL* glyoxylate carboxyligase, *TSR* tartronic semialdehyde reductase. The enzymes and reactions of the photosynthesis and photorespiration are shown in *black*, and the introduced photorespiratory bypass is shown in *red*. Adapted from [21]

Photorespiration is caused by the oxygenase activity of the CO_2_-fixing enzyme ribulose-1,5-bisphosphate carboxylase/oxygenase (RuBisCO). In C3 plants such as camelina, RuBisCO catalyzes the reaction of CO_2_ with the five carbon sugar phosphate ribulose-1,5-bisphosphate (RUBP) about 75 % of the time to generate two molecules of 3-phosphoglycerate (3-PGA). The other 25 % of the time, RuBisCO catalyzes the reaction of O_2_ with RUBP [11–13] to generate one molecule of 3-PGA and one molecule of phosphoglycolate (2-PG) (Fig. 1). 3-PGA is used in the chloroplast for RUBP regeneration or is diverted towards starch or sucrose synthesis. 2-PG is a toxic metabolite which inhibits photosynthesis in the chloroplast [14]. To avoid this detrimental effect, 2-PG is converted to glycolate, which is transported to the peroxisomes, and via photorespiration, the two molecules of 2-PG eventually form one molecule of 3-PGA at the loss of one molecule of CO_2_ through mitochondria. Therefore, while the carboxylase activity of RuBisCO leads to carbon fixation, the oxygenase activity causes carbon loss. In addition, each round of photorespiration generates one molecule each of H_2_O_2_ and NH_3_, both of which are toxic and require energy for their metabolism. In terms of the energy cost, photorespiration increases the cost of photosynthetic carbon fixation by about 50 % [15].

While photorespiration seems to be a wasteful process regarding carbon and energy losses, the current knowledge suggests that photorespiration is important for plant survival, and may provide reducing power for nitrate assimilation [16–20]. Mutants in photorespiratory pathway genes are often lethal [16–20]. Synthetic metabolic pathways that bypass photorespiration, but do not eliminate it, have been shown to reduce photorespiratory carbon loss and thereby increase photosynthetic efficiency and biomass yield [21–23]. The most effective of these different photorespiratory bypasses is the one described by the Peterhansel group [21, 24]. Kebeish and coworkers introduced three enzymes from the *Escherichia coli* glycolate catabolic pathway into *Arabidopsis thaliana* chloroplasts to create this photorespiratory bypass [21]. In this pathway (Fig. 1), glycolate was oxidized to glyoxylate in the chloroplasts by the bacterial glycolate dehydrogenase (GDH). Then, two molecules of the 2-carbon glyoxylate were converted by glyoxylate carboxyligase (GCL) to a three-carbon tartronic semialdehyde (TSA) releasing one CO_2_ inside the chloroplast. The tartronic semialdehyde is converted to glycerate by the enzyme tartronic semialdehyde reductase (TSR) to re-enter photosynthesis. There are several advantages to this approach. First, it generates a competing pathway for the plant photorespiratory cycle, as shown by the reduced glycine/serine ratios in transgenic plants expressing the bypass genes. Second, the CO_2_ from glyoxylate is released directly inside the chloroplasts for re-fixation by RuBisCO. Expression of the photorespiratory bypass genes resulted in significant increases in photosynthetic CO_2_-fixation and consequently biomass accumulation in *Arabidopsis* [21].

In addition to the full bypass, partial bypass comprising of GDH alone was also found to be successful in increasing photosynthesis and biomass production in *Arabidopsis* [21] and potato [23]. GDH expression increases glyoxylate content in chloroplasts [21]. Independent reports from literature indicate that isolated wild-type chloroplasts from various species can metabolize glycolate as well as glyoxylate to release CO_2_ [21, 25, 26], but the related enzymes are not yet identified. While there is evidence that in isolated chloroplasts, glyoxylate can reduce or even inhibit photosynthesis by inhibiting RuBisCO activation [27, 28], excess glyoxylate in leaf disks was found to promote photosynthesis and reduce photorespiration [29, 30]. In an attempt to investigate methods to reduce photorespiration and increase photosynthetic productivity, Oliver and Zeleitch found that tobacco leaf disks floated on 5–25 mM potassium glyoxylate (pH 4.6) showed doubling of ^14^CO_2_ fixation by photosynthesis [30], reduction in CO_2_ evolution in light by 57 % and reduction in glycolate concentration by 40–60 %. These results indicated that glyoxylate acts to increase photosynthesis primarily by decreasing glycolate formation and photorespiration [30]. In another report, incubating soybean mesophyll cells with glyoxylate increased photosynthetic CO_2_ fixation by 150 %, and inhibited CO_2_ incorporation in glycolate and glycine by 71 % [29]. While the mechanism of this inhibition is unknown, GDH overexpression in chloroplasts of partial and full bypass expressing plants reduced photorespiration, increased photosynthesis and increased biomass fixation in *Arabidopsis* by 80 % [21] and in potato by up to 131 % [23].

We introduced this photorespiratory bypass into camelina to increase photosynthetic efficiency and decrease CO_2_ loss by photorespiration. We introduced either a partial bypass (GDH only; DEF2 lines) or the full bypass (GDH, GCL and TSR; DEF2+TG1 lines) into camelina with the enzymes targeted to the chloroplasts. Transgenic plants expressing both partial and full bypass genes had reduced levels of photorespiration, increased photosynthesis rates and increased vegetative biomass. The research presented here indicates that the expression of photorespiratory bypass is a successful approach towards increasing crop productivity and seed yield in the biofuel crop camelina.

## Results

### Introduction of photorespiratory bypass genes into *Camelina sativa*

**Fig. 2:**
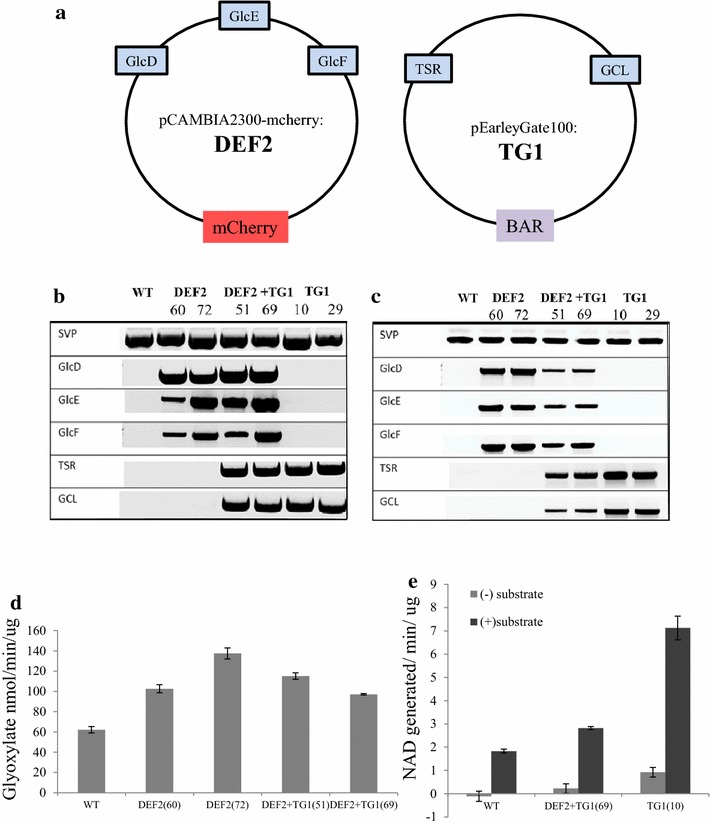
Expression of photorespiratory bypass genes in camelina.** a** Constructs were generated for introducing photorespiratory bypass genes into *Camelina*. Each coding sequence was preceded by a constitutive promoter and fused to a chloroplast transit peptide sequence. The DEF2 construct contains GlcD, GlcE and GlcF sequences cloned into a modified pCAMBIA2300-mCherry vector where NPTII from pCAMBIA2300 has been replaced by the mCherry gene. The TG1 construct contains the GCL and TSR sequences cloned into the pEG100 vector. Plants were either transformed with the DEF2 construct alone, TG1 construct alone, or co-transformed with DEF2 and TG1.** b** Plants passing selection were tested for gene insertion using PCR of gDNA. Primers (Additional file 1: Table S1) were used to amplify the endogenous reference gene *SVP1*(+) and transgenes GlcD, GlcE, GlcF, TSR and GCL.** c** The expression of transgene mRNAs was tested by semi-quantitative RT-PCR using the same primers as above.** d** The proteins GlcD, GlcE and GlcF combine to form the glycolate dehydrogenase (GDH) enzyme complex. The activity of GDH was tested using isolated chloroplasts from transgenic and WT plants.** e** The activity of enzymes TSR and GCL was evaluated in a coupled NADH depletion assay. Using sodium glyoxylate as substrate, NAD generation in chloroplast extracts was compared

For the catabolic conversion of glycolate to glycerate and CO_2_ in chloroplasts (photorespiratory bypass, Fig. 1), genes encoding three bacterial enzymes—GDH, GCL and TSR were introduced into *C. sativa* nuclear genome and the proteins were targeted to chloroplasts. The bacterial sequences were codon-optimized for expressing in plants (GenBank: KP967458-KP967462). The first step of the photorespiratory bypass is the oxidation of glycolate to glyoxylate. This step is catalyzed by the enzyme glycolate dehydrogenase (GDH), containing three subunits, GlcD, GlcE and GlcF (construct DEF2, Fig. 2a). The second step involves ligation of two glyoxylate molecules to form a 3-carbon tartronic semialdehyde (TSA) and the release of a CO_2_ molecule. This conversion is catalyzed by glyoxylate carboxyligase (GCL). The third step involves conversion of TSA to glycerate by tartronic semialdehyde reductase (TSR). The gene constructs of GCL and TSR are included in vector TG1 (Fig. 2a). Plants were either transformed with DEF2 (DEF2 lines; partial bypass), TG1 (TG1 lines) or co-transformed with DEF2 and TG1 constructs together (DEF2+TG1 lines; full bypass). The integration and expression of the transgenes in plants was verified by PCR using gDNA (Fig. 2b) and cDNA templates (Fig. 2c). Using PCR, we were able to isolate homozygous lines for all the three combinations—DEF2, TG1 and DEF2+TG1. In T_3_ homozygous plants, the protein expression of the transgenes was quantified by ELISA with antibodies specific to each protein. Green leaves of 3-week-old plants were used to quantify bypass proteins. On average, in DEF2 transgenics, GlcD expression was detected to be 0.76 µg/g fresh weight (FW), GlcE was 0.61 µg/g FW, and GlcF was 0.89 µg/g FW. On average, in TG1 plants, the expression of GCL was 5.7 µg/g FW and TSR was 11.8 µg/g FW (Additional file 1: Table S3). The catalytic activity of the bypass enzymes was evaluated by isolating the chloroplasts from transgenic and WT plants, and using chloroplast extracts to perform enzymatic assays. The activity of GDH was determined by measuring glyoxylate generation after feeding chloroplast extracts with glycolate [31]. Chloroplasts from DEF2 and DEF2+TG1 transgenics showed 65–120 % higher GDH activity than WT chloroplasts (Fig. 2d). The activities of enzymes GCL and TSR were evaluated in a coupled NADH depletion assay [32]. Chloroplasts from TG1 and DEF2+TG1 transgenics showed 50–300 % higher GCL+TSR activity than WT chloroplasts (Fig. 2e).

These data indicate that the bypass enzymes were active in the chloroplasts of transgenic camelina lines. Due to visibly improved phenotypes of DEF2 and DEF2+TG1 lines, homozygous plants from representative lines transformed with these constructs were used further for phenotypic and biochemical analysis.

### Bypass plants have reduced photorespiration and increased photosynthesis

**Fig. 3:**
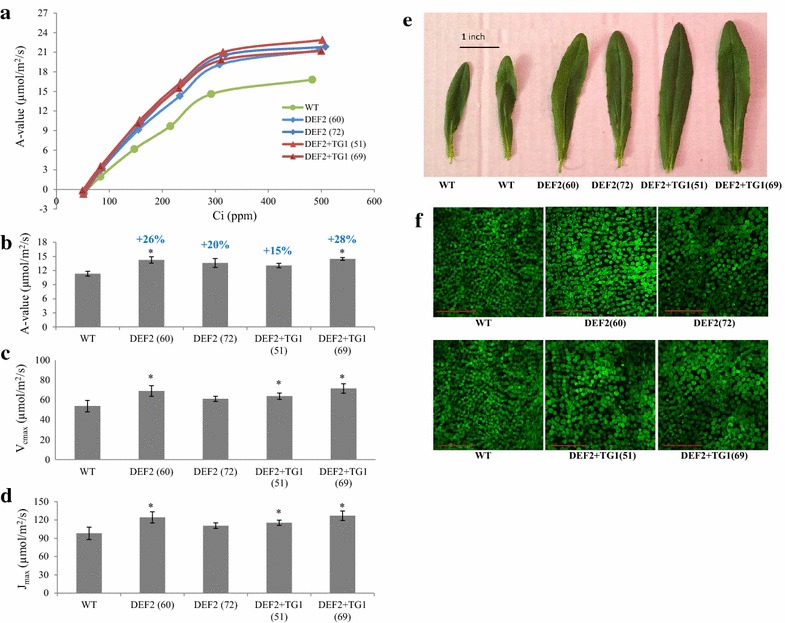
Gas exchange measurements and anatomical features of leaves of bypass transgenics. **a** *A/C*_i_ *curves* were generated at saturating light (1500 PAR) using the youngest fully expanded leaf. The *A*/*C*_i_ curves were used to calculate *V*_cmax_ (maximum rate of RuBiSCO-mediated carboxylation) (**c**) and *J*_max_ (maximum rate of electron transport) (**d**) in transgenic and WT leaves.** b** Apparent rate of CO_2_ fixation rate (A-value µmol/m^2^/s) of representative plants from two independent DEF2, DEF2+TG1 and WT lines was measured using LI6400-XT at chamber light levels(~440 PAR). On average, DEF2 transgenics had 20–26 % and DEF2+TG1 plants had 15–28 % increase in photosynthesis over WT plants (*n* = 4, *p* < 0.05; *error bars* standard error).** e** The leaves on transgenic plants were larger than WT. The twelfth leaf of 7-week-old plants was harvested and approximate leaf area was compared. On average, DEF2 transgenics had about 50 % and DEF2+TG1 plants had about 75 % increase in leaf area over WT plants (*n* = 4, *p* < 0.05).** f** The leaf 12 from representative plants was cleared, and the cross-section was examined for cell distribution. The palisade mesophyll cells and the intercellular spaces were larger the leaves of the bypass leaves (*scale bar* 0.5 mm)

During photorespiration, glycolate forms glyoxylate which is transaminated to glycine [33], which in turn reacts with folate to form serine (Fig. 1). The amount of glycine and serine in leaves is indicative of the carbon flux through photorespiration [34]; higher ratios of glycine/serine are related to higher RUBP oxygenation [21, 35]. We investigated the glycine/serine ratio in leaves of DEF2 and DEF2+TG1 transgenics. Samples were taken in photorespiratory conditions (high light, about 5 h after dawn; day samples), and non-photorespiratory conditions (1 h before dawn; night samples). In the day samples, glycine/serine ratio was reduced by 23 % in DEF2 and 27 % in DEF2+TG1 plants. There were no differences in glycine/serine ratio between the night time samples. We further investigated whether the bypass-related CO_2_ evolution in chloroplasts improved plant photosynthesis. CO_2_ fixation rates (*A*) were measured in leaves of WT, DEF2 and DEF2+TG1 plants at different intercellular CO_2_ concentrations (*C*_i_) in high light (1500 PAR) to generate *A*/*C*_i_ curves using LI-6400XT. At low CO_2_ levels starting from about 100 *C*_i_ (ppm), DEF2 and DEF2+TG1 had higher rates of photosynthetic CO_2_ fixation compared to WT (Fig. 3a). At ambient CO_2_ and light (~400 ppm, 430 PAR), 4-week-old DEF2 plants showed 20–25 % and DEF2+TG1 plants showed 14–28 % increase in CO_2_ fixation over WT per unit leaf area (Fig. 3b). The data from *A*/*C*_i_ curves were used to calculate *V*_cmax_ (maximum rate of RuBiSCO-mediated carboxylation) and *J*_max_ (maximum rate of electron transport) for multiple WT, DEF2 and DEF2+TG1 plants using the equations by von Caemmerer, Farquhar and Sharkey [36, 37]. In DEF2 line 60 and DEF2+TG1 lines 51 and 69, there was a significant increase in both *V*_cmax_ and *J*_max_ (Fig. 3c, d). To determine if the increased photosynthesis was due in part to increased photosynthetic efficiency of photosystem II, we measured the chlorophyll fluorescence in dark-adapted (pre-dawn) plants. The *F*v/*F*m ratios (Additional file 1: Figure S2) showed no significant change in photosystem efficiency between transgenic plants and WT.

While the increase in photosynthetic rate per unit area was notable, DEF2 and DEF2+TG1 plants also developed substantially larger leaves compared to WT. The leaf area of the twelfth leaf on 7-week-old plants was measured. On average, the leaf area at this developmental stage was 11.4 cm^2^ in WT, 14.8 cm^2^ in DEF2 plants and 16.9 cm^2^ in DEF2+TG1 transgenic plants, representing increases of 30 % (DEF2) and 48 % (DEF2+TG1) over WT (Fig. 3e; Additional file 1: Table S4). Because of this dramatic increase in leaf size, we undertook an anatomical examination of transgenic and WT leaves. Microscopic evaluation showed that DEF2 and DEF2+TG1 plants had larger mesophyll cells, with larger intercellular spaces than WT (Fig. 3f). In a 1200 µm^2^ leaf area photographed under 20× objective, there were about 750 ± 20 cells in WT leaves, but only 484 ± 20 and 494 ± 40 cells in the DEF2 and DEF2+TG1 plants, respectively. This increased cell size in the transgenic lines (as estimated by the cell number per 1200 µm^2^) would account for a leaf area increase of 55 % (DEF2) and 52 % (DEF2 +TG1), respectively. Given the average observed increases in leaf area of 30–48 %, it suggests that the increase in leaf area in the transgenic lines compared to WT does not involve increase in cell division, but may fully be explained by increased cell size. Despite fewer and larger cells in transgenic leaves, the chlorophyll content per gram fresh leaf was found to be similar to WT (Additional file 1: Table S1).

### Bypass expressing plants have a growth advantage over WT

**Fig. 4:**
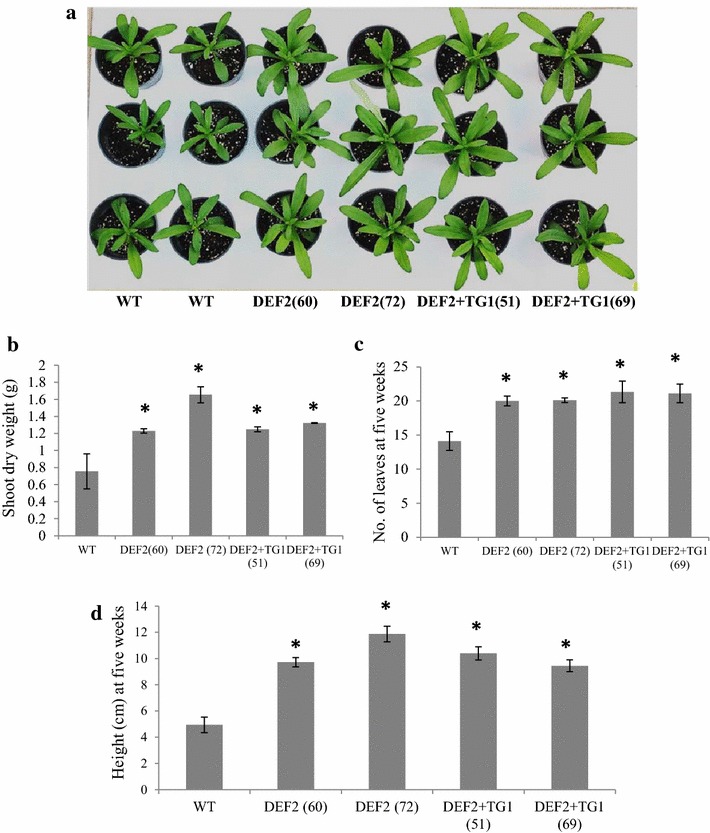
Enhanced biomass fixation of bypass transgenics. **a** Five-week-old representative bypass transgenics show improvement in growth over WT plants.** b** In 5-week-old plants, the above-ground dry weight increased by 62–118 % over WT in DEF2 transgenics and 65–75 % over WT in DEF2+TG1 transgenics (*n* = 3, Student’s *t* test, *p* value ≤0.05).** c**,** d** In 5-week-old plants, DEF2 and DEF2+TG1 transgenics grew about 5 inches taller than WT and formed about three more leaves per plant. By week 7, DEF2 and DEF2+TG1 transgenics grew about 8 inches taller than WT and had about five more leaves (n = 9) *Error bars* standard error

Bypass expressing plants showed a significant increase in biomass accumulation compared to WT (Fig. 4a). The shoot dry weight of DEF2 plants was on average 62 % (line 60) and 118 % (line 72) higher than WT (Fig. 4b). Full bypass expressing lines (DEF2+TG1) had on average a 65 % (line 51) and 75 % (line 69) increase in shoot dry weight compared to WT. At 7 weeks of age, DEF2 and DEF2+TG1 plants grew at least 15 cm taller and had 4 or 5 more leaves than WT plants (Fig. 4c, d). This phenotype was consistent among multiple independent transgenic lines of DEF2 and DEF2+TG1 constructs. Overall, out of twelve independent insertion lines of DEF2, eight showed more than 5 cm of height advantage over WT at about 6 weeks after germination. For the DEF2+TG1 construct, out of 10 lines tested, nine lines had more than 5 cm of height advantage over WT at about 6 weeks after germination.

**Fig. 5:**
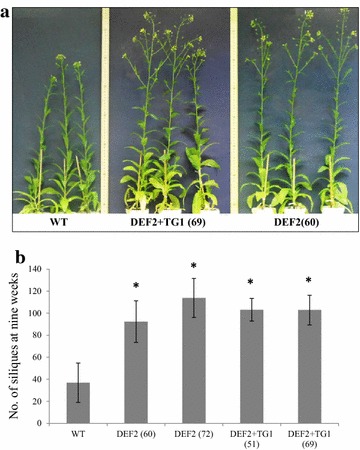
Earlier floral induction of bypass transgenics. **a** Bypass transgenics flowered about a week earlier than WT controls. At 7 weeks of age, bypass transgenics start setting seeds when WT plants only start flowering.** b** Bypass transgenics had a higher number of siliques than WT. By week 9, bypass transgenics had over 2.5 times more siliques than WT on average. (*n* = 9) *Error bars* standard error

Another prominent feature of bypass transgenics was earlier floral induction. Under short-day conditions (9 h day), bypass expressing plants flowered about 1–2 weeks earlier than WT plants. By the time WT plants flowered (6 weeks of age), DEF2 and DEF2+TG1 transgenics started producing siliques (seed pods) (Fig. 5a). By week 9, DEF2 and DEF2+TG1 transgenics had over 2.5 times the number of siliques on WT plants (Fig. 5b). The development of new siliques continued in WT while seed maturation was underway in DEF2 and DEF2+TG1 plants. Overall, bypass plants generated more siliques compared to WT and the seeds were mature for harvesting 1–2 weeks earlier than WT.

### Bypass expressing plants have increased seed yield per plant

**Fig. 6:**
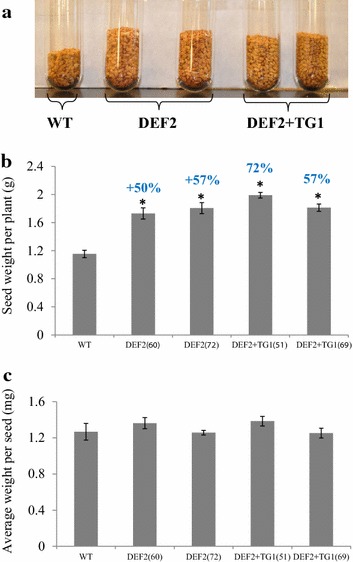
Increased whole plant seed yields of bypass transgenics.** a** Seed yield of representative plants from DEF2, DEF2+TG1 and WT lines was measured. Bypass transgenics have substantially higher seed yield than WT.** b** In short-day conditions, DEF2 plants have 50–57 % higher seed yield and DEF2+TG1 plants have 57–72 % higher seed yield than WT (*n* = 9, Student’s *t* test *p* < 0.05). **c** The weight per seed of bypass transgenics is comparable to the weight per seed of WT plants (*n* = 6 sets, 50 seeds per set)

Bypass expressing plants showed higher seed yields compared to the control plants. DEF2 lines had a 50–57 % increase and DEF2+TG1 lines had a 57–72 % increase in seed yield over WT plants (Fig. 6a, b). The increase in yield is consistent through multiple generations of homozygous transgenics. When groups of 50 seeds were weighed for each line, no significant difference in the average weight per seed was observed between transgenic and WT plants (Fig. 6c). The number of seeds per pod between transgenic and WT plants was also not significantly different. Thus, the increased yields were due to increased number of siliques on the transgenics.

### Bypass plants have increased seed oil production and comparable meal characteristics

**Fig. 7:**
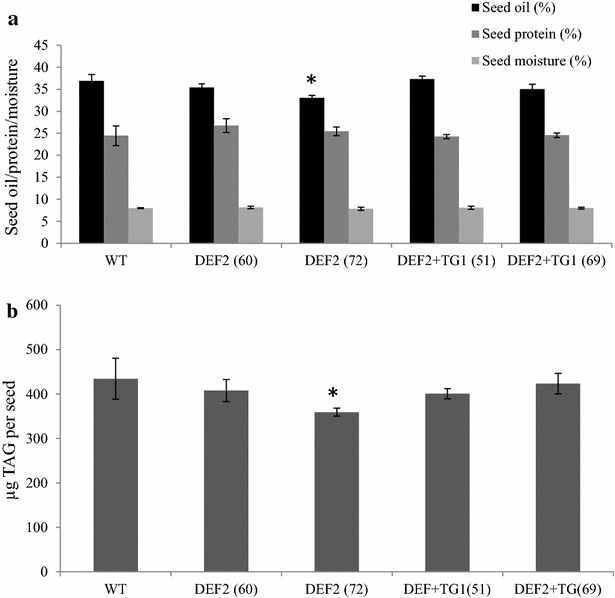
Oil, protein and moisture contents of bypass transgenics.** a** NMR analysis on seed extracts showed that bypass transgenics had similar total oil, total protein (soluble and insoluble) and total moisture content as WT (3 replicates per plant, 1.5 g seed each replicate).** b** Using gas chromatography, the TAG content for different bypass transgenic seed was measured (50 seeds per sample, six replicates per plant). The TAG content in bypass transgenics per seed is comparable to WT

To evaluate the quality of seeds from transgenic plants, we analyzed the oil, protein and moisture contents by NMR. Overall, bypass expressing plants had similar oil, protein and moisture contents compared to WT (Fig. 7a). Seed triacylglycerides (TAG) content was also measured using gas chromatography, and the results were very similar to the oil content data obtained by NMR (Fig. 7b). The DEF2 line 72 was found to have lower oil content per seed weight compared to other bypass lines and WT. However, all other independent DEF2 and DEF2+TG1 lines had oil content comparable to WT plants on a per-seed basis.

### Bypass plants show indication of increased CO_2_ use efficiency

**Fig. 8:**
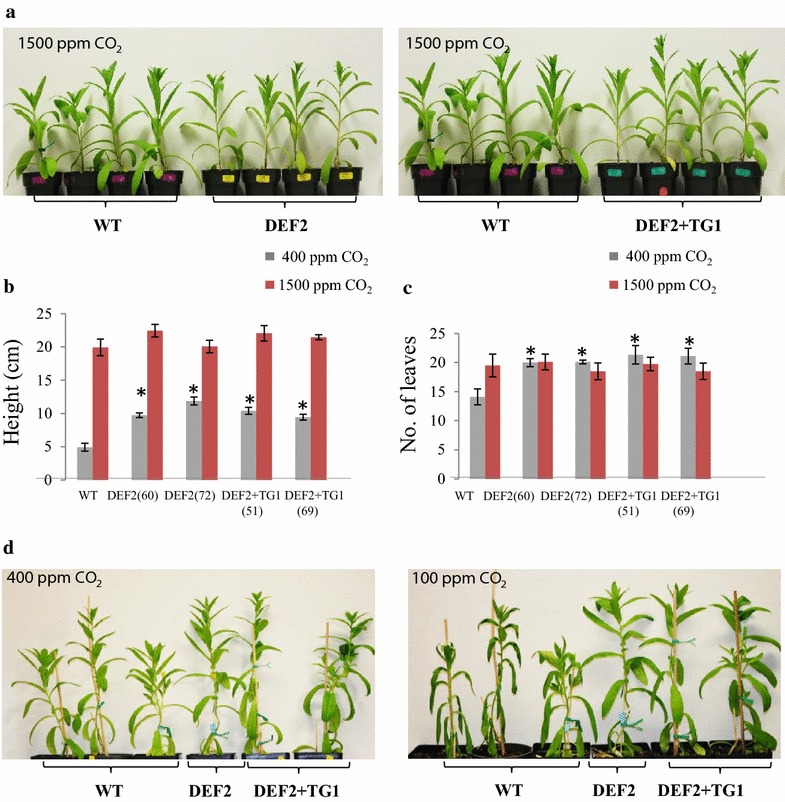
Phenotypes of bypass transgenics when grown in elevated or reduced CO_2_ environment.** a** In elevated CO_2_ conditions (1500 ppm), bypass plants have no detectable growth advantage over WT plants.** b**,** c** At 5 weeks of age, bypass transgenics grown in high CO_2_ showed no significant height or leaf number advantage over WT (*dark red bars*). This is in contrast with the significant advantage (marked by *asterisk*) in height and leaf number observed in bypass transgenics in ambient CO_2_ conditions (*light gray bars*).** d** When plants are grown in ambient CO_2_ and exposed to low CO_2_ conditions (100 ppm) for 5 h, WT show a shriveling response, which is not shown by the bypass transgenics. 10 ≤ *n* ≤ 12, *error bars* standard error

To test if the increase in growth in the transgenic plants was due to higher CO_2_ use efficiency, we measured the growth of bypass expressing plants under ambient CO_2_, high CO_2_ and low CO_2_ conditions. At 5 weeks of age, when grown in ambient CO_2_ conditions, transgenic plants were about 4–7 cm taller than WT plants, but at elevated CO_2_ (1500 ppm), the heights of the transgenic plants were similar to WT (Fig. 8a, b). Similarly, in ambient CO_2_ conditions, bypass plants had 5–7 leaves more than WT, but at elevated CO_2_ conditions, there was no significant difference in the leaf numbers (Fig. 8c). We further tested if there was an effect of low CO_2_ on the growth of transgenics. When the plants were grown for 5 weeks in ambient CO_2_ and then moved to low CO_2_ conditions (~100 ppm) for 5 h, we observed a dramatic phenotypic difference between transgenic and WT plants. Under this treatment, the WT plants became wilted, whereas the DEF2 and DEF2+TG1 plants remained turgid (Fig. 8d). Upon restoration of ambient CO_2_ conditions, WT plants reverted back to a turgid phenotype. These data indicate that CO_2_ supply may have improved in bypass transgenic plants.

### Bypass plants have increased rate of dark respiration and higher starch accumulation

**Fig. 9:**
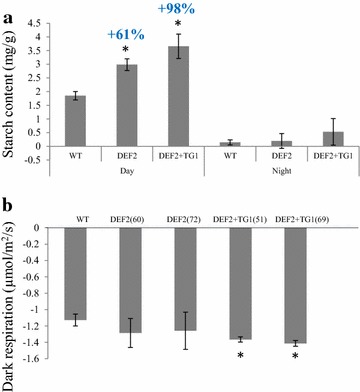
Diurnal starch accumulation and respiration rates of bypass transgenics.** a** Starch content was measured in leaves isolated 1 h *before* dawn (night samples) and 5 h *after* dawn (day samples). In the day samples, bypass plants have higher mg/g starch content than WT. (*n* = 4, *error bars* standard error).** b** Bypass plants have higher respiration than WT (pre-dawn). LI6400-XT was used to measure leaf respiration (*n* = 3, *error bars* standard error)

Assimilated CO_2_ in leaves may be allocated in various ways: biosynthesis of cellular materials for growth and maintenance such as sugars or lipids, exported via the phloem to sink tissues, or stored as energy and carbon source for the dark phase in form of carbohydrates. Because bypass expressing plants showed increased photosynthetic CO_2_ fixation rates, we analyzed diurnal sugar and starch accumulation in their leaves. Sucrose was quantified by LC–MS from extracts of 5-week-old plants grown in ambient CO_2_ 5 h after dawn. We did not observe any significant differences in sucrose accumulation in the bypass expressing lines compared to WT. Starch accumulation was quantified from extracts of 5-week-old plants grown in ambient CO_2_ and harvested 1 h before dawn (night samples) or 5 h after dawn (day samples). We observed that in the day samples, starch accumulation increased by about 60 % in DEF2 plants, and nearly doubled in DEF2+TG1 plants (Fig. 9a). In the night samples, no significant change in the starch content was observed in transgenic plants.

We also investigated the rate of respiration of bypass plants in pre-dawn conditions. About 2 h before dawn, respiration rate was analyzed using LICOR on 8-week-old plants (plants were already forming siliques). DEF2 plants had slightly, and DEF2+TG1 plants had significantly increased rates of respiration compared to WT (Fig. 9b).

### Global changes in transcript abundances correlated with the expression of the photorespiratory bypass

**Fig. 10:**
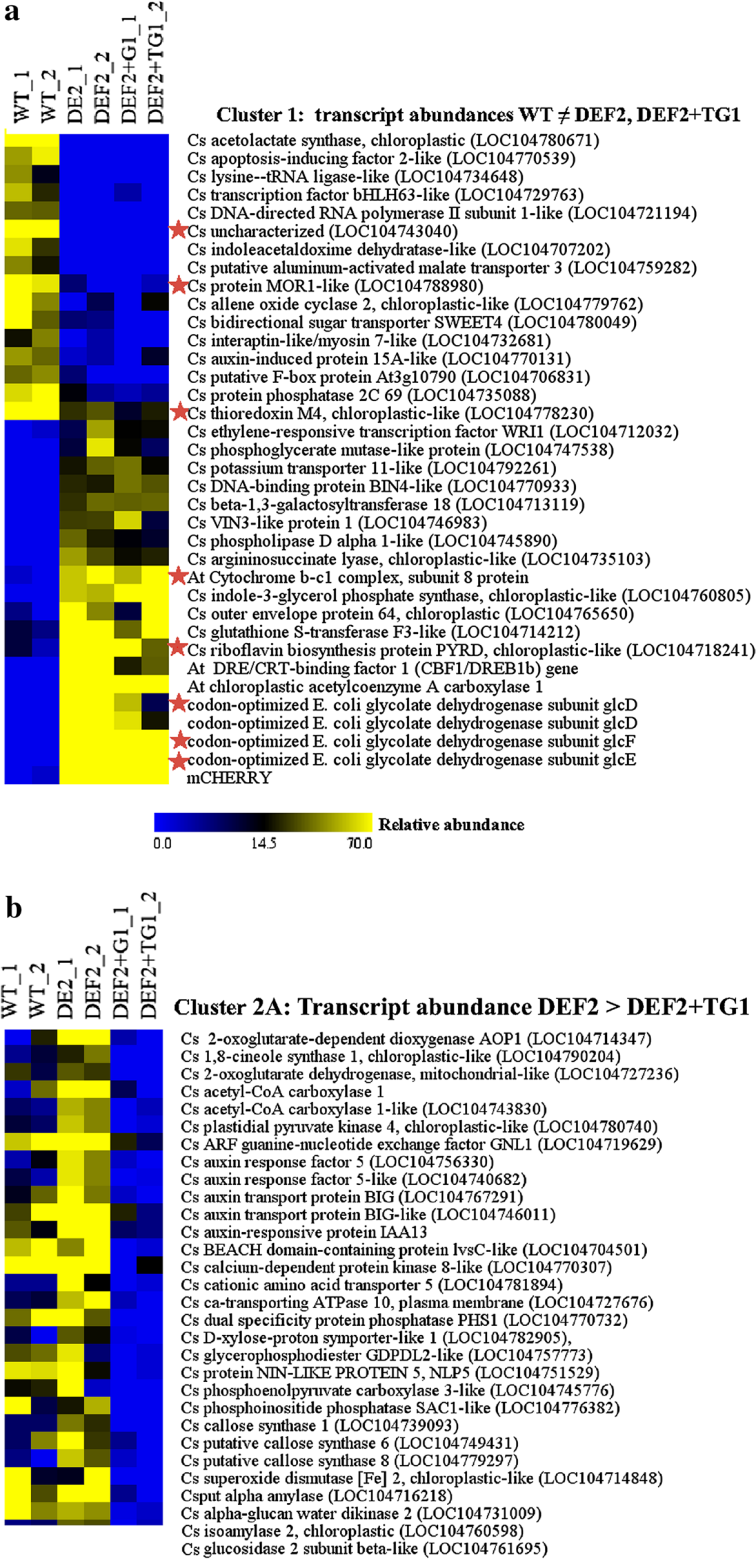
Selected differentially expressed transcripts in leaf tissue of WT, DEF, and DEF2+TG1 bypass lines were clustered based on expression pattern or function.** a** Cluster 1 shows transcripts that were differentially expressed between WT and the lines expressing either the DEF2 or DEF2+TG1 constructs.** b** Cluster 2A shows transcripts that are more highly expressed in leaves of DEF2 lines compared to DEF2+TG1 bypass lines. The full data set is included in Additional file 1: Table S2 (*blue* low; *yellow* high; linear range)

To evaluate any changes in gene expression that correlate with the increased photosynthetic CO_2_ assimilation and growth rate of bypass expressing plants, we performed RNAseq analysis on leaf samples from 7-week-old DEF2, DEF2+TG1, and WT plants with two biological replicates and two technical replicates per sample. To visualize the pattern of differentially expressed genes in the samples (at least twofold), plotSmear plots were generated using the edgeR package (Additional file 1: Figure S3). These plots show the relationship between the contig count concentrations and the fold changes in their relative expression in log scale. Significant changes were observed in gene expression patterns between the bypass samples and WT, and also between the DEF2 and DEF2+TG1 samples. Pairwise comparison between different lines using the MEV analysis tool [38] showed construct-specific differences (Fig. 10a). Out of the total of 128,578 quantified unique loci (transcripts) that were annotated against the NCBI database (*e* < 10^−6^), 587 transcripts were differentially expressed between DEF2 vs. WT, while more than twice as many, 1247 genes were differentially expressed between DEF2+TG1 vs. WT. About 290 contigs showed differential expression between DEF2 and DEF2+TG1 plants vs. WT. About 828 contigs were differentially expressed between DEF2 and DEF2+TG1 plants. Smaller groups of loci were expressed either only in DEF2 (50 contigs, expressed in DEF2 but absent in DEF2+TG1 and WT) or only in DEF2+TG1 (269 contigs, expressed in DEF+TG1 but absent in DEF2 and WT). The full table of sequencing results and differential expression is provided in Additional file 2.

**Fig. 11:**
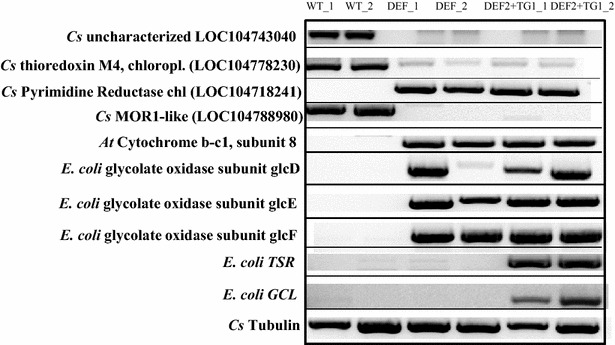
Select transcripts that were shown to be differentially expressed in Fig. 10a were analyzed for confirmation using semi-quantitative PCR. These transcripts were marked *star* in Fig. 10a

The highest transcript abundance differences between the lines were found to be the transgenes and selection marker mCherry (Fig. 10a). GlcD, GlcE and GlcF transcripts were only found in both transgenic lines and not in WT, while TSR and GCL transcripts were only found in the transcriptome of DEG2+TG1 plants. This indicated that our transcriptome analysis reflected the actual transcriptome profiles in the different plant lines. To further validate our transcriptome data, we amplified the transgenes and other selected differentially expressed transcripts (marked by red star in Fig. 10a), with gene-specific primers using RT-PCR (Fig. 11). Here too, expression of the transgenes was only detected in the respective lines, and the expression patterns of the selected genes matched those seen in the sequencing results. With this validation of the sequencing data, we identified the differentially expressed transcripts (loci) to distinguish endogenous camelina genes that were significantly affected in their transcript abundance by expression of the bypass transgenes (for full data set see Additional file 2).

We annotated the differentially expressed transcripts and sorted them into clusters based on their expression patterns. Cluster 1 shows transcripts that are similar in both transgenic lines (DEF2 and DEF2+TG1), but different in WT (Fig. 10a). The abundance of these transcripts (112 total; Additional file 2) was either elevated (higher in transgenics: 75 genes) or repressed (higher in WT: 37) due to the expression of the bypass genes. This cluster contains the transgenes from *E. coli* glycolate dehydrogenase (DEF) and the marker-gene mCherry. Some of the amino acid biosynthesis pathway enzymes including acetolactate synthase (isoleucine biosynthesis), indoleacetaldoxime dehydratase (tryptophan biosynthesis) were lower in the bypass expressing plants, while elements of the respiratory electron transport chain (cytochrome b-c1) and fatty acid/lipid metabolism (plastidic acetyl-CoA carboxylase), arginine biosynthesis (argininosuccinate lyase) and indole-3-glycerol phosphate synthase (tryptophane biosynthesis), riboflavin biosynthesis genes (plastidic pyrimidine reductase; PYRD) had higher abundance in both bypass expressing lines compared to WT (Fig. 10a).

**Fig. 12:**
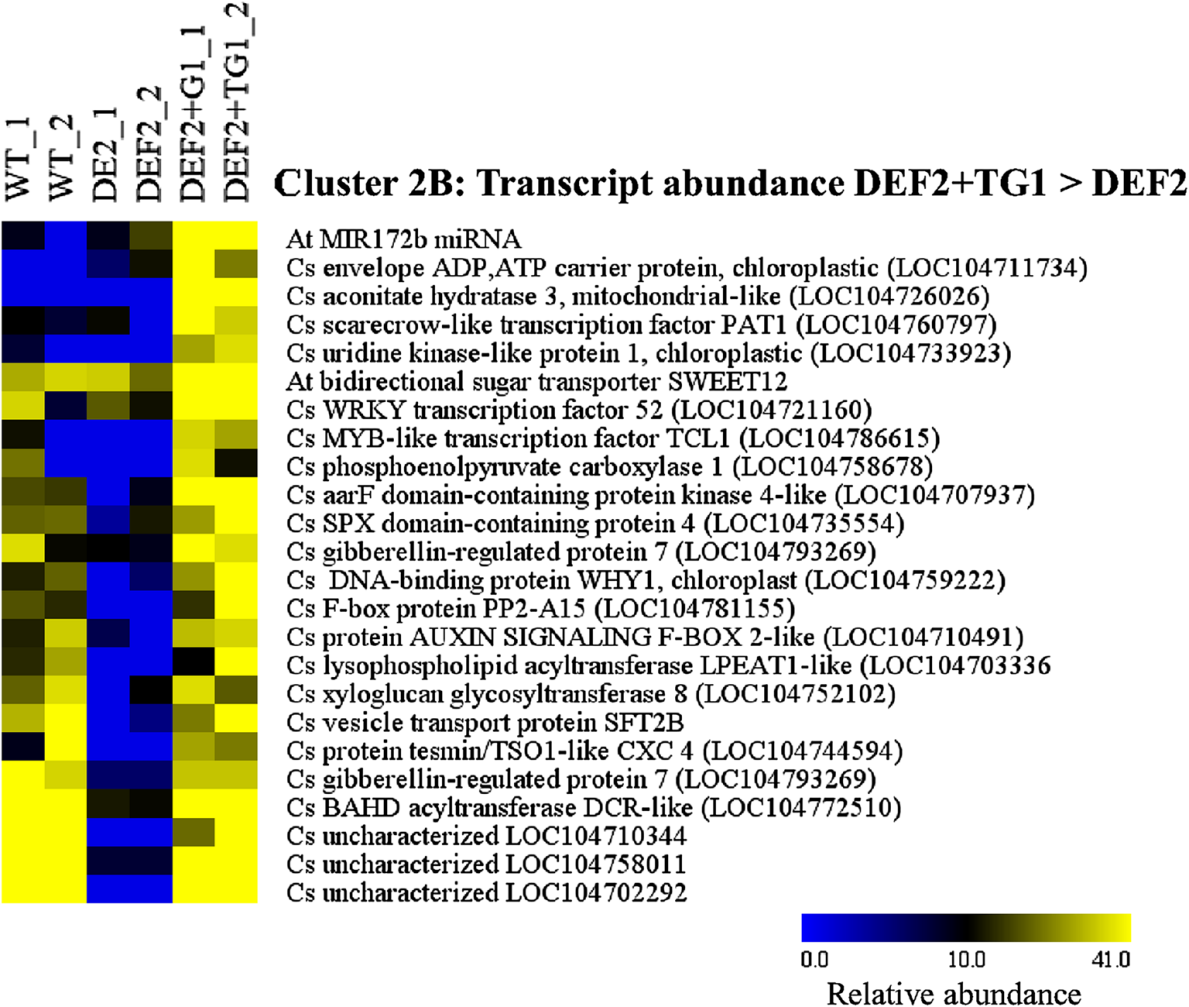
Cluster 2B shows transcripts with higher expression levels in DEF2+TG1 compared to DEF lines. The full data set is included in Additional file 1: Table S2 (*blue* low; *yellow* high; linear range)

The other interesting transcripts were those that showed differences in abundance between the two transgenic lines expressing either only glycolate dehydrogenase (DEF2) of the full bypass (Full) lines. Cluster 2A shows transcripts with higher abundance in DEF2 lines compared to Full bypass expressing lines (Fig. 12, Additional file 1: Table S2). This cluster is enriched in auxin signaling transcripts (ARF5, ARF5-like, IAA13, auxin transport protein BIG and BIG-like), starch metabolism transcripts (alpha amylase, isoamylase, glucan:water dikinase), several callose synthase isoforms, as well as two almost identical cytoplasmic acetyl-CoA carboxylase 1 transcripts encoding ACCases involved in flavonoid biosynthesis. A chloroplast-like superoxide dismutase also had higher abundance in DEF2 and WT compared to DEF2+TG1 plants.

Transcripts in cluster 2B show higher transcript abundance in the full bypass expressing lines compared to the lines only expressing the glycolate dehydrogenase (DEF2). This cluster could be further categorized with respect to the transcript levels in WT tissues. MiRNA173b which promotes photoperiodic flowering and regulates the transitions between developmental stages and in specifying floral organ identity in Arabidopsis is higher in Full bypass lines compared to WT and DEF2 lines. A similar pattern is seen on the chloroplastic ATP/ADP carrier in the envelope membrane, the mitochondrial aconitase and the scarecrow-like transcription factor PAT1, involved in phytochrome A-mediated signal transduction (Fig. 12, Additional file 1: Table S2). Most transcripts in this cluster which showed similar expression in WT and Full bypass lines, but lower expression in DEF2 lines were of uncharacterized function. A BAHD acyltransferase required for cutin polyester formation and seed hydration as well as a gibberellin-regulated protein, two F-Box transcription factors, vesicle-transport proteins and a phospholipid biosynthesis gene also showed this pattern of transcript abundances between the transgenic lines.

## Discussion

To enhance the potential of camelina as a biofuel crop, increasing its seed yield is a critical step. By introducing a partial or full photorespiratory bypass, we increased seed yield in camelina by 50–72 %. We also observed that bypass expressing plants had other agriculturally important traits such as earlier floral induction (by 1 week) and earlier seed maturation (by 1–2 weeks). Introducing the photorespiratory bypass may be a valid approach to increase yields in C3 plants in general, a claim strengthened by previous reports of *Arabidopsis* plants expressing full and partial bypass [21], and potato plants expressing partial bypass [23].

Compared to these reports [21, 23], we noted some similarities and some novel differences in camelina. Consistent with previous reports, expressing the photorespiratory bypass in camelina improved photosynthetic efficiency (Fig. 3) and biomass fixation (Fig. 4). Expression of the partial bypass in potato plants increased the tuber size, weight and the tubers were set about a week earlier than WT plants [23]. While seed yield data from *Arabidopsis* are not available [21], in camelina, expression of both partial and full bypass increased seed yield, number of siliques formed per plant and the transgenics flowered about a week earlier than WT (Fig. 5). The reduced glycine/serine pools in transgenic plants indicate that the photorespiratory carbon flux has also been reduced. However, further investigation is required to ascertain which aspect of the photorespiratory pathway is affected. First, biochemical models of leaf photosynthesis can be used to determine the CO_2_ partial pressure in WT, DEF2 and DEF2+TG1 chloroplasts at which the RuBisCO carboxylation rate approaches the rate of CO_2_ loss by photorespiration (CO_2_ photocompensation point). [39, 40]. In *Arabidopsis,* Kebeish et al. [21] observed that CO_2_ compensation points were reduced both in partial and full bypass lines. The gas exchange studies may also be supplemented with quantitative analysis of photorespiratory intermediates, as summarized by [41–43]. Further, it would be of interest to measure the pool sizes of photorespiratory intermediates in low CO_2_ [21] as well as high CO_2_ growth conditions.

Similar to *Arabidopsis* [21], the bypass-related phenotypic traits in camelina were not observed in elevated CO_2_ conditions (Fig. 8a–c), when the rate of photorespiration is low. We further observed that under low CO_2_ (100 ppm, Fig. 8d), WT plants wilted, while the transgenic lines remained turgid. Such a phenotype may be caused by a difference in stomatal aperture: WT had to keep the aperture fully open to gain more CO_2_ but lost more water at the same time, while the transgenic plants did not have to do so due to a higher CO_2_ concentration inside the chloroplasts [44]. In DEF2+TG1 plants, high chloroplastic CO_2_ levels can be attributed to the enzyme GCL, which converts glyoxylate into tartronic semialdehyde in the chloroplasts and releases CO_2_ as a result. However, it remains to be elucidated why DEF2 plants, which only express the GDH enzyme, show a similar phenotype.

The increased glyoxylate generation in DEF2 plants can either be transported to peroxisomes for processing or it may be catabolized within the chloroplasts. While the pathways involved in the plastid metabolism of glyoxylate are not yet identified, previous research indicates that isolated WT chloroplasts can metabolize glyoxylate to release CO_2_ [25, 26]. Blume et al. [45] observed that pyruvate can inhibit glyoxylate-dependent CO_2_ evolution. Because pyruvate and glyoxylate have similar chemical structures, they may compete for the binding site of the plastidic pyruvate dehydrogenase complex (PDC) [45]. PDC has thus been cited as one of the enzymes that may be able to decarboxylate glyoxylate in the chloroplasts for CO_2_ evolution [45, 46]. However, this or related pathways of glycolate and/or glyoxylate catabolism in chloroplasts may not exclusively account for the increased photosynthetic productivity of GDH-expressing plants. In leaf disks, excess glyoxylate was found to increase ^14^CO_2_ fixation by 1.2–2.3 fold, and decrease glycolate concentration and photorespiratory CO_2_ evolution by more than 50 % [30]. It is possible that GDH-expressing plants, such as the DEF2 plants in this report and in the literature [21, 23] take advantage of these unknown pathways and exhibit increased photosynthesis and reduced photorespiration by a two-faceted approach in the chloroplast competing for glycolate as a substrate to form glyoxylate, and a glyoxylate inhibition of glycolate production [29, 30]. While the alternate pathway for glyoxylate catabolism is not well understood, it seems to increase chloroplast CO_2_ levels in DEF2 chloroplasts and increase photosynthesis, just as GCL seems to increase CO_2_ levels in DEF2+TG1 chloroplasts [21]. In agreement with this hypothesis, bypass transgenics show growth phenotypes conditional on the CO_2_ concentration of the air in which they grow.

Previous research has shown that plants grown in elevated CO_2_ conditions have increased respiration [47, 48]. In soybean plants grown in elevated CO_2_ (560 ppm), Leakey and coworkers reported that photosynthesis increased by about 20 %, generating more photosynthetic storage products such as starch. They also reported increased dark respiration and postulated that it was a mechanism to increase starch degradation at night, which prevented excess starch accumulation in leaves at dawn [48]. Bypass expressing camelina plants showed a similar phenotype as the “high-CO_2_” grown soybean plants. The accumulation of starch in the light period was more than twice that of WT camelina plants and was completely utilized by the end of the dark phase. Bypass expressing plants also showed higher pre-dawn respiration rates (Fig. 9b). Consistent with this observation, in our sequencing data, expression of respiration-related genes was affected. For example, the respiration complex cytochrome b-c1 was expressed at higher levels in the bypass expressing plants compared to WT plants (Fig. 10a). The transcript profiles suggest that the accumulation of starch in the DEF2+TG1 lines was most likely due to a reduction of starch turnover during the light. Transcripts of starch degrading enzymes such as glucan:water dikinase and isoamylase were significantly lower in the DEF2+TG1 lines compared to WT. The higher starch levels in the leaves of the bypass plants could indicate increased internode elongation during the dark phase. The elevated respiration is fueled by starch degradation and provides the energy (ATP) for sucrose export to the phloem. Sucrose export costs account for about 30 % of the dark respiration rate in mature leaves [49], so in our fully grown camelina leaves, the increased amounts of starch and correlating increases in dark respiration suggest increased sucrose export via the phloem during the night—again correlating with increased growth rates during the dark phase.

Because the full bypass and the partial bypass with the alternate pathway may both release CO_2_ in chloroplasts, but may generate different intermediate and end products, we observe similarities as well as differences in phenotypes and transcript abundances between DEF2 and DEF2+TG1 plants. Genes that are affected in their transcript abundance between the DEF2 plants and DEF2+TG1 transgenics could offer a clue to potential pathways and players involved. Although several of those genes are not yet characterized (Figs. 10a, b, 12), transcripts encoding enzymes functional in terpene biosynthesis (cineole-synthase = geranyl-diphosphate diphosphate-lyase; and cytosolic ACCases) as well as enzymes involved in plastidic fatty acid biosynthesis (pyruvate kinase, plastid ACCase) suggest a possible allocation of assimilated carbon through plastidic lipid metabolism in DEF2+TG1 plants. There was increased transcript abundance for multiple sugar transporter genes in DEF2 and DEF2+TG1 plants (Fig. 10b). Accumulation of sucrose in source cells can cause feedback inhibition of photosynthesis [50–53]. Overexpression of sugar transporters in bypass transgenics may have increased the efficiency of export of sugars (sucrose) via phloem to sink tissues. This could help in increasing seed yields, as well as further improving photosynthetic efficiency by relieving the feedback inhibition of photosynthesis [54]. As a corollary to that, transgenic overexpression of known sugar transporters may further remove sucrose build-up, and may increase photosynthesis even more in bypass transgenics.

An interesting difference in the results from our study compared to the previously reported effects of plants grown in high CO_2_ [55, 56] or plants expressing photorespiratory bypasses using bacterial [21] or plant genes [22] is the change in leaf phenotype. Previous studies with plants expressing photorespiratory bypasses reported development of larger, lighter colored and thinner leaves in transgenic *Arabidopsis* lines [21, 22]. In contrast, bypass transgenics in camelina had larger leaves and similar chlorophyll content as WT leaves (Fig. 3e, Additional file 1: Figure S1). While the leaves were larger, they were not noticeably thinner. In the mesophyll of bypass leaves, the cells were more loosely arranged, with fewer but larger mesophyll cells per unit area than WT leaves which had more tightly packed, smaller mesophyll cells (Fig. 3f). One possible reason for this difference in leaf anatomy could be species related. Another could be developmental constraints on the number of cells formed within the leaf, or an extended cell elongation stage. Indeed, the difference between the cellular distribution in bypass transgenics compared to WT is reminiscent of older leaves near the end of elongation stage compared with younger leaves [57]. As leaves expand, chloroplast surface area also increases [57]. Further, RuBisCO levels have been reported to correlate with total chloroplast volume [58–61], and leaf photosynthesis per unit area correlates linearly with increasing RuBisCO [58]. Analysis of whether larger cells in bypass transgenics have a higher number of chloroplasts, whether the chloroplasts are larger in size, and measurements of overall RuBisCO content would be necessary to correlate the anatomical data with increased photosynthesis per unit area in the bypass plants. It also remains to be investigated whether the larger leaves in camelina bypass transgenics shade lower leaves and prevent them from reaching their highest photosynthetic potential in field conditions [62].

Another area that must be investigated in future studies is the nitrogen use efficiency (NUE) of bypass transgenics. Because the photorespiratory bypass reduces carbon flux through photorespiration, it also reduces the release of ammonia from glycine catabolism and re-fixation into glutamine. Therefore, plants expressing the photorespiratory bypass are predicted to have an increase of 15 % in NUE, although this has never been tested [24, 63, 64]. NUE is defined as the proportion of all nitrogen inputs that are accumulated by the plant in biomass and seed, as well as nitrogen that is recycled by plant residue into the soil [65]. NUE of a plant affects both the amount of nitrogen fertilizer applied, as well as potential recycling of nitrogen into the soil. In Arkansas rice fields, it was observed that about 18 % of variable productions costs could be attributed to nitrogen fertilizer and its application, making it one of the largest expenses in the grain production [66]. Therefore, NUE is an important feature for optimization of ‘benefit to cost ratio’ of a crop plant [65], and may be an advantage of bypass plants. An increase in leaf nitrogen content can cause the formation of larger chloroplasts [58] while increasing *V*_cmax_ [67, 68] and further increasing photosynthesis.

## Conclusion

Camelina is a biofuel crop with a great potential. By increasing photosynthetic productivity, the strategy of photorespiratory bypass could have a significant economic impact for camelina production, by not only increasing seed oil and meal harvested per acre of land, but also by reducing crop cycle time and thereby reducing input resources of land and labor. Because photorespiration is a cause of carbon loss in most C3 plants, this strategy may be applied to other agriculturally important C3 crops, such as rice and soybean. The aspect of increasing nitrogen use efficiency is one that if realized in bypass expressing plants as predicted, would lead to significant reduction in fertilizer application, and lower the environmental cost of agriculture.

## Methods

### Gene synthesis, optimization and cloning

The photorespiratory bypass genes used in the experiments originated from the *E. coli* K-12 genome (strain MG1655) available on the NCBI website (gi49175990). Among them, *GDH* has three subunits encoded by three genes, *GlcD*, *GlcE*, and *GlcF*, while *GCL* and *TSR* are encoded by single genes [20]. The coding sequences were synthesized using codons optimized genes for expression in *Arabidopsis* [69] by GenScript (GenScript, Piscataway, NJ, USA) (GenBank: KP967458-KP967462). The CaMV 35S promoter [70] was selected to drive the expression of *GlcE*, *GCL* and *TSR*. The tobacco *EntCUP4* promoter [71] was chosen to drive expression of *GlcD*, and the *Arabidopsis ACTIN2* promoter [72] was selected to drive the expression of *GlcF*. The *Arabidopsis* RuBisCO small subunit (RBCS) transit peptide [73] was selected for chloroplast targeting of GlcD, GlcF and TSR, while the biotin carboxyl carrier protein (BCCP) transit peptide [73] was chosen for chloroplast targeting of GlcE and GCL. The three individual gene constructs for GDH were cloned into the multiple cloning site of a modified pCAMBIA2300 binary vector (CAMBIA Institute http://www.cambia.org/), in which the *NPTII* gene for kanamycin-resistance selection in plants was replaced by the *mCherry* coding sequence from the plasmid ER-RB [74, 75]. The resulting vector is called DEF2 (Fig. 2a). The GCL and TSR coding sequences were synthesized with flanking *att* sites and cloned in the destination binary vector pEarleyGate100 (pEG100) via gateway-assisted recombination [76], resulting in vector TG1 with a *bar* selectable marker gene [77, 78] (Fig. 2a).

### Transformation procedure and selection of transgenics

The DEF2 and TG1 binary vectors were individually introduced into *Agrobacterium* strain GV3101 for transformation of *C. sativa var. Calena*. Six-week-old camelina plants were transformed with either DEF2 or TG1 or co-transformed with both DEF2 and TG1 constructs using the floral dip method as described [79] with vacuum infiltration at 20 psi for 5 min. For co-transformation, cultures of *Agrobacterium* containing TG1 and those containing DEF2 were mixed together in equal concentrations and used to transform camelina plants in an approach modified from [80]. DEF2 transformants were screened at the seed stage (T1) for mCherry fluorescence under a fluorescence dissecting microscope using 560 nm excitation wavelength. TG1 transgenic plants were selected by spraying 3-day-old seedlings (T1) with herbicide Finale^®^ (diluted to 0.045 % phosphinothricin).

### PCR analysis of transgenic plants

To verify the presence of the transgenes in the selection positive plants, PCR was performed on genomic DNA of the selected plants. DNA was isolated using the DNeasy plant mini kit (Qiagen, Valencia, CA, USA). To investigate the expression of the transgenes, RNA was isolated from leaves of transgenic and WT plants using the Trizol reagent (Life Technologies, Grand Island, NY). cDNA was synthesized using the SuperScript III cDNA construction kit (Life Technologies) [81]. The primers used for amplification of the genes utilized are: GlcD: RBCS-F1 and GLCD-R2; GlcE: BCCP-F1 and GLCE-R2; GlcF: RBCS-F1 and GLCF-R2; GCL: BCCP-F1 and GCL-R2; TSR: RBCS-F1 and TSR-R2. Camelina *SVP*-*like 1* gene (GenBank AY177710) primers, SVP1 FP and SVP1 RP, were used as a positive control. Primer sequences and sizes of their amplification products are given in Additional file 1: Table S6.

### Plant growth conditions

Plants were grown under short-day conditions (9 h light/15 h dark cycle). Light intensity was 430 μmol m^−2^ s^−1^ during the day and the temperature was 20 °C at both day and night. CO_2_ conditions were provided by a custom-modified growth chamber (Caron Inc., Marietta, OH, USA). A CO_2_ cylinder and an ambient air cylinder connected with a scrubbing apparatus were attached to the chamber through solenoid valves. The exact CO_2_ conditions desired were maintained by either infusing CO_2_ into the chamber, or infusing air scrubbed through soda lime which removes CO_2_ from the air.

### Plants used in the experimental assays

Most of the plants used in the experimental assays were from the T3 or T4 generation of homozygous lines expressing DEF2 (lines 60 and 72) or expressing both, DEF2+TG1 (lines 51 and 69). Homozygosity was determined by examining segregation ratios of fluorescence or Finale resistance of at least 48 seeds or plants per line. Individual plants were further checked by PCR and RT-PCR to confirm transgene presence and expression before they were used for further experiments. For other lines when offspring plants were still segregating, only PCR positive plants were selected.

### Phenotypic analysis and dry weight measurements

To investigate and record phenotype changes, plants were monitored and photographed weekly. The heights of the plants were measured using ImageJ [82]. The date of flowering was noted to observe the age of plants at flowering. The number of siliques formed per week was counted from each plant until the plants cease to form more siliques. The seeds were harvested when plants were mature and the seed pods were dry. The seeds were cleaned using a sieve. Seeds harvested from each plant were weighed. For shoot dry weight measurements, 5-week-old plants were harvested from the ground level and the shoots were dried at 60 °C for 14 days before weighing.

### Protein quantification by ELISA

Three-week-old plants were used for ELISA assays to evaluate the abundance of proteins encoded by the transgenes. Affinity-purified polyclonal antibodies were custom-ordered from GenScript. The antibodies were raised in rabbits against peptide antigens of GlcD, GlcE, GlcF, GCL and TSR, respectively. The antigen sequences are provided in Additional file 1: Table S6. The secondary antibody was goat anti-rabbit IgG [HRP] (GenScript). Leaf samples (0.8–1.5 g) were harvested from WT and transgenics and ground using a pestle in 2 mL 1× PBS. A hundred µL of this extract was directly pipetted into a 96-well microplate. For calibration, a dilution series of the target peptide was placed in the extract of 1.5 g WT leaf tissue ground in 1X PBS buffer. The samples were incubated overnight at 4 °C and then at 37 °C for 30 min. The supernatants were then discarded, and the plate was washed three times with 1× PBST, followed by primary antibody and secondary antibody incubations. Each well was finally probed with 100 µl of 1× TMB substrate (Thermo Fisher Scientific, Rockford, IL, USA). The color reaction was allowed to develop for 1–15 min, and terminated by addition of 25 µl of 5 N HCl. The optical density was measured using a Synergy™ HT microplate reader (BioTek, Winooski, VT, USA). The standard curve was made using the optical densities of known peptide concentrations, and it was used to measure the amount of target peptide in different plant lines.

### Enzyme activity assays

To examine enzymatic activities of the various transgenes, intact chloroplasts were isolated and chloroplast intactness was verified as described by [83]. The purity of the chloroplasts was checked for contamination by peroxisomes by testing for catalase activity [84]. About 8 g of leaf material was used per sample to isolate chloroplasts. In the final step of chloroplast isolation [83], EDTA and MgCl_2_ were excluded from the wash buffer, since EDTA inhibits TSR activity [85]. Thirty µl of isolated chloroplasts was lysed with 600 µl of 0.1 % Triton X-100. The chloroplast extract was centrifuged at 4 °C and the supernatant was assayed for protein quantity by Bradford assay. The activity of glycolate dehydrogenase was tested in DEF2 and DEF2+TG1-expressing plants using the assay as described in [31]. The experiment was scaled down to use chloroplast extracts containing 25 µg total protein in each 800 µl reaction. Glyoxylate generation was measured by change in O.D. (324 nm) due to the formation of glyoxylate phenylhydrazone (extinction coefficient 1.68 × 10^4^ M^−1^ cm^−1^). The activity of GCL and TSR was measured in a coupled assay adapted from [32]. Chloroplast extracts containing 10 µg total protein each were added to six wells per sample in a 96-well microplate as replicates. The volume of samples in each well was brought up to 50 µl using the chloroplast lysis buffer. Twenty-five µl of 20 mM NADH was added to each well, and the O.D. (340 nm) was measured using the HT microplate reader. In three replicates per sample, 25 µl of 0.2 M sodium glyoxylate (substrate) was added, and the O.D. (340 nm) was measured every minute for a period of 45 min. Using the differences in O.D. before and after adding sodium glyoxylate (delta O.D.) and using an NADH concentration gradient standard curve, the NADH depletion due to NAD generation in the presence of sodium glyoxylate was measured.

### Gas exchange measurements

The gas exchange measurements were made using LI-6400XT (LI-COR Biosciences, Lincoln, NE, USA) instrument and software. For generating *A*/*C*_i_ curves, the youngest fully expanded leaves of 3- to 4-week-old plants were clamped to the LI-6400XT gas exchange chamber. The light source was maintained at 1500 PAR and leaf temperatures were set at values closest to ambient (~20 °C). The manufacturer’s auto-program was used to collect measurements to generate the A/C_i_ curves. Readings were logged after leaf was stabilized at 400 ppm CO_2_, followed by gradually decreasing chamber CO_2_ to 300, 200, 100, and 50 ppm; increasing again for two readings at 400 ppm and collecting one final reading at 600 ppm, completing eight total readings per leaf sample. Values for *A* (net CO_2_ assimilation rate) and *C*_i_ (intercellular CO_2_ concentration, calculated using equations of [36]) were obtained from the instrument to generate *A*/*C*_i_ curves. The values of *A* and *C*_i_ from five representative curves per plant line were used to solve for *V*_cmax_ and *J*_max_ using the *A*/*C*_i_ curve fitting utility and equations of [37]. For photosynthetic rate determination at 400 ppm, 4- to 5-week-old plants were used. Photosynthetic rate readings were taken about 3 h after dawn. The light source was adjusted to be identical to the PAR detected in the plant growth chamber (~430 PAR). About 8-week-old plants were used for respiration rate measurements. Respiration readings were taken 2 h before dawn. Faint green light was used as a light source to help load samples into the LI-6400XT chamber followed by a 2-min long dark acclimation in the chamber. For both photosynthetic and respiration measurement, the reference CO_2_ was set at 400 ppm (ambient) and the growth chamber temperature (~22 °C) was maintained. Developmentally similar leaves were selected from each plant and each leaf was allowed to acclimate to the CO_2_ exchange chamber of the instrument for 2 min, after which the reading for gas exchange was recorded.

### Leaf staining and confocal microscopy

To gain insights into cell sizes in the transgenic leaves, leaf number 12 of 7-week-old camelina plants was harvested. The leaf was put in a clearing solution of 1 % SDS and 200 mM NaOH for 3–5 days, after which they were washed and stained with Congo-Red (0.33 mg/ml) overnight. The stained leaves were washed with the clearing solution, and the length of the leaf was measured. Leaf disks were cut out exactly from the center, exceeding no more than 2 mm in width. The disks were mounted on glass slides using 10 % glycerol as mounting medium. The disks were photographed [86] and imaged using Leica Laser Scanning confocal microscope using 488 nm excitation laser, 602–297 nm filters, a 39 µm pinhole and a 10× objective.

### Chlorophyll content measurement

Leaves were harvested from WT, DEF2, and DEF2+TG1 plants (3 replicates each). Fresh weight of each sample was recorded (about 100 mg). Leaves were then frozen in liquid nitrogen and ground into fine powder. Pigments were subsequently extracted in 5 mL of 80 % acetone [87]. Each extract was centrifuged at 16,000x*g*, the supernatants saved, and the resulting pellet containing pigments was extracted three more times with equal volumes of acetone (5 mL each) until the green color disappeared. All the acetone phases from a sample were combined and kept on ice in the dark. One at a time, the chlorophyll extracts were quickly measured in the spectrophotometer at 663, 646, and 710 nm wavelengths. The content of chlorophyll (*a* + *b*) was determined using the following equation: Chlorophyll (*a* + *b*) = 7.18 × (A663–A710) + 17.32 × (A646–A710) [87].

### Metabolic profiling and starch quantification

Leaves were collected from about 4-week-old plants grown under short-day conditions. Samples were collected at two distinct diurnal time points—1 h before (pre-dawn, night samples) and 5 h into the day period (day samples). Leaves of the same physiological age (fourth leaf from the top) that appeared green and actively photosynthesizing were harvested, weighed and immediately frozen in liquid N_2_. The leaves were ground in liquid N_2_ using mortar and pestle, and the soluble metabolites were immediately extracted twice in a water:methanol:chloroform solution (1:2.5:1) (adapted from [88]). For each extraction, the samples were mixed into 1 ml solution by inversion for 1 h at 4 °C and centrifuged for 15 min at 4 °C. The insoluble fraction was collected for starch extraction. The soluble fraction was further phase-separated using chloroform and water [89]. The hydrophilic phase hence obtained was analyzed by LC–MS. For solvent A2, 970 µl water, 2.4 ml tributylamine, 30 ml methanol and 859 µl glacial acetic acid were used. For solvent B2, LC–MS grade methanol was used. The metabolites were separated using Synergi 2.5u Hydro RP 100 A column (100 × 2 mm) column using Agilent 6200 Series Q-TOF LC/MS (Agilent, CA, USA). The serine and glycine peaks per isolated, and the peak areas were normalized by leaf fresh weight. The serine and glycine (ng/ml) contents were calculated using standard curves generated for pure serine and glycine. For starch quantification, the insoluble fraction was dried in speed vacuum for 1 min, then solubilized in 0.2 N KOH for 4 h at 80 °C, sonicated for 10 min and digested in triplicate reactions for 16 h at 37 °C in 4 volumes of digestion buffer [50 mM sodium acetate, 5 U/mL amylase, 5 U/mL amyloglucosidase (Sigma-Aldrich, MO), pH 4.8]. The digested starch was assayed for glucose by conversion with glucose-6-phosphate dehydrogenase as previously described [90].

### Seed composition analysis

The lipid, protein and moisture contents were analyzed in the WT and the transgenic DEF2 and DEF2+TG1 seeds using NMR at the Seed Laboratory at Oregon State University (http://seedlab.oregonstate.edu/oil-protein-and-moisture-determination-seeds-using-nmr). In addition, seed oil content was also evaluated by gas chromatography following the method of [91] with minor modification. Per sample, fifty seeds were used for measuring oil content and fatty acid profiles. Six replicates were included per line. Three hundred μg of triheptadecanoin was used as a TAG internal standard. One ml of 2.5 % (v/v) sulfuric acid in methanol was added to each sample and kept at 90 °C for 90 min. The fatty acid methyl ester (FAME) extracts were analyzed by GC using an Agilent 6890 gas chromatograph with flame ionization detection. Resolution of FAMEs was achieved with an HP-INNOWax column (30 m in length, 0.25 mm in inner diameter). Assuming most of the fatty acids are in TAGs in camelina seeds, total fatty acids were measured as methyl esters of (approximately) the total oil for comparison. The calculation of oil content from GC analysis was done as described previously [92].

### Transcriptome profiling

Transcript abundances were analyzed from WT and one line each of DEF2 and DEF2+TG1 plants using RNAseq. Two developmentally comparable leaves from 7-week-old plants were harvested from two plants per line. The leaves were immediately frozen and ground in liquid nitrogen and RNA was isolated using Trizol [81]. RNA concentration and quality was assessed using a spectrophotometer (Nanodrop) and an Agilent Bioanalyzer RNA Eukaryote Nanochip. Three µg of total RNA at a concentration of ≥2000 ng/ml, and RNA Integrity Number ≥8 were used for cDNA library preparation. The cDNA library was constructed using 2 kits: the Dynabeads mRNA direct micro kit for polyA mRNA extraction from total RNA and the Ion Total RNA-Seq kit v2 for Whole Transcriptome Library preparation (Life Technologies). The libraries of all samples were sequenced as single-end 100 bp read length using the Ion Proton platform on P1 chips (Life Technologies). In total, 172 million reads were generated from the sequencing platform. The quality of the individual sample reads was assessed using the FastQC program (http://www.bioinformatics.babraham.ac.uk/projects/fastqc/). All reads were quality filtered at Q20 Phred score. Low-quality reads were trimmed 15 nt from 3′ end of reads. Finally, identical sequences were removed from individual samples using the FAST-X toolkit (http://hannonlab.cshl.edu/fastx_toolkit/). All processed reads were pooled together and assembled de novo with Trinity software [93]. The resulting set of transcripts was annotated against different databases, i.e., *C. sativa, Arabidopsis thaliana*, *E. coli* K12 MG1655, UniprotKB/Swiss-Prot and NR database using the Blastx tool to obtain high-quality matches (*e* value <10 × *e*^−6^ over at least 90 % of the query sequence) and the BLAST2GO software [94]. The cleaned reads were mapped against the annotated de novo assembly using the BWA aligner tool [95]. Based on number of transcripts aligned to the assembly, transcript abundance was measured using the eXpress tool (http://bio.math.berkeley.edu/eXpress/). These transcript counts were used as inputs to the EdgeR package in the R programming language software to determine differentially expressed transcripts between various categories. To display the differential gene expression between the genotypes, plotSmear plots were generated, and differentially expressed genes were clustered using Microarray Software Suite MeV TM4 (http://www.tm4.org/mev.html) [96]. RT-PCR validation of RNAseq data was conducted for selected genes. RNA was isolated from representative WT and transgenic plants. cDNA synthesis was conducted using SuperScriptIII (Life Sciences) cDNA synthesis kit. Primers used for validation are listed in Additional file 1: Table S6.

### Statistical analysis

For phenotypic comparative analyses, 3–9 biological replicates and 2 technical replicates were used for each plant line. Two-tailed Student’s *t* test procedures were used to compare means of transgenic vs. WT sample statistics. The significance level was set at 5 %.

## Additional files

10.1186/s13068-015-0357-1 Supplementary tables and figures. **Figure S1.** Chlorophyll content of bypass transgenics. The chlorophyll content per leaf fresh weight was measured. Overall, bypass transgenics had comparable amount of chlorophyll to WT per mg. leaf tissue. **Figure S2.** Fv/Fm ratios of bypass transgenics. Chlorophyll fluorescence (Fv/Fm) was measured using LI6400-XT on dark-acclimated plants. Based on the ratios, the maximum efficiency of photosystem II was estimated to be comparable between bypass transgenics and WT. **Table S1.** The transgenic protein content in various lines was determined using peptide-specific antibodies by ELISA. **Table S2.** Leaf area of different transgenic lines (measured using ImageJ). **Figure S3.** (a-c) plotSmear plots were generated using the edgeR package. A plotSmear plot is used to visualize the relationship between the contig count concentrations and fold change in a log scale. In the figure, red dots represent differentially expressed genes between the samples. The orange dots represent contigs with zero count values in some of the samples. The blue line represents biological significance at 2 log-FC. **Table S3.** Primer and antigen sequences.
10.1186/s13068-015-0357-1 Bypass RNA seq data. Excel file containing RNA sequencing data from DEF2, DEF2+TG1 and WT lines.

## Abbreviations

WT: wild-type
DEF2: transgenic plant or construct with the three subunits of glycolate dehydrogenase (GlcD, GlcE and GlcF)
TG1: transgenic plant or construct with the two genes- tartronic semialdehyde reductase and glyoxylate carboxyligase
DEF2+TG1: transgenic plant expressing DEF2 construct with TG1 construct
*SVP*: *Camelina sativa* SVP-like protein gene
