# Supplementary material for: A photorespiratory bypass increases plant growth and seed yield in biofuel crop Camelinasativa
Source: Biotechnol Biofuels. 2015 Oct 29;8:175. doi: 10.1186/s13068-015-0357-1 (PMC4625952; doi:10.1186/s13068-015-0357-1)
Supplement: Supplementary file 1 — 10.1186/s13068-015-0357-1 Supplementary tables and figures. Figure S1. Chlorophyll content of bypass transgenics. The chlorophyll content per leaf fresh weight was measured. Overall, bypass transgenics had comparable amount of chlorophyll to WT per mg. leaf tissue. Figure S2. Fv/Fm ratios of bypass transgenics. Chlorophyll fluorescence (Fv/Fm) was measured using LI6400-XT on dark-acclimated plants. Based on the ratios, the maximum efficiency of photosystem II was estimated to be comparable between bypass transgenics and WT. Table S1. The transgenic protein content in various lines was determined using peptide-specific antibodies by ELISA. Table S2. Leaf area of different transgenic lines (measured using ImageJ). Figure S3. (a-c) plotSmear plots were generated using the edgeR package. A plotSmear plot is used to visualize the relationship between the contig count concentrations and fold change in a log scale. In the figure, red dots represent differentially expressed genes between the samples. The orange dots represent contigs with zero count values in some of the samples. The blue line represents biological significance at 2 log-FC. Table S3. Primer and antigen sequences. [file 13068_2015_357_MOESM1_ESM.pptx]

## Slide 1
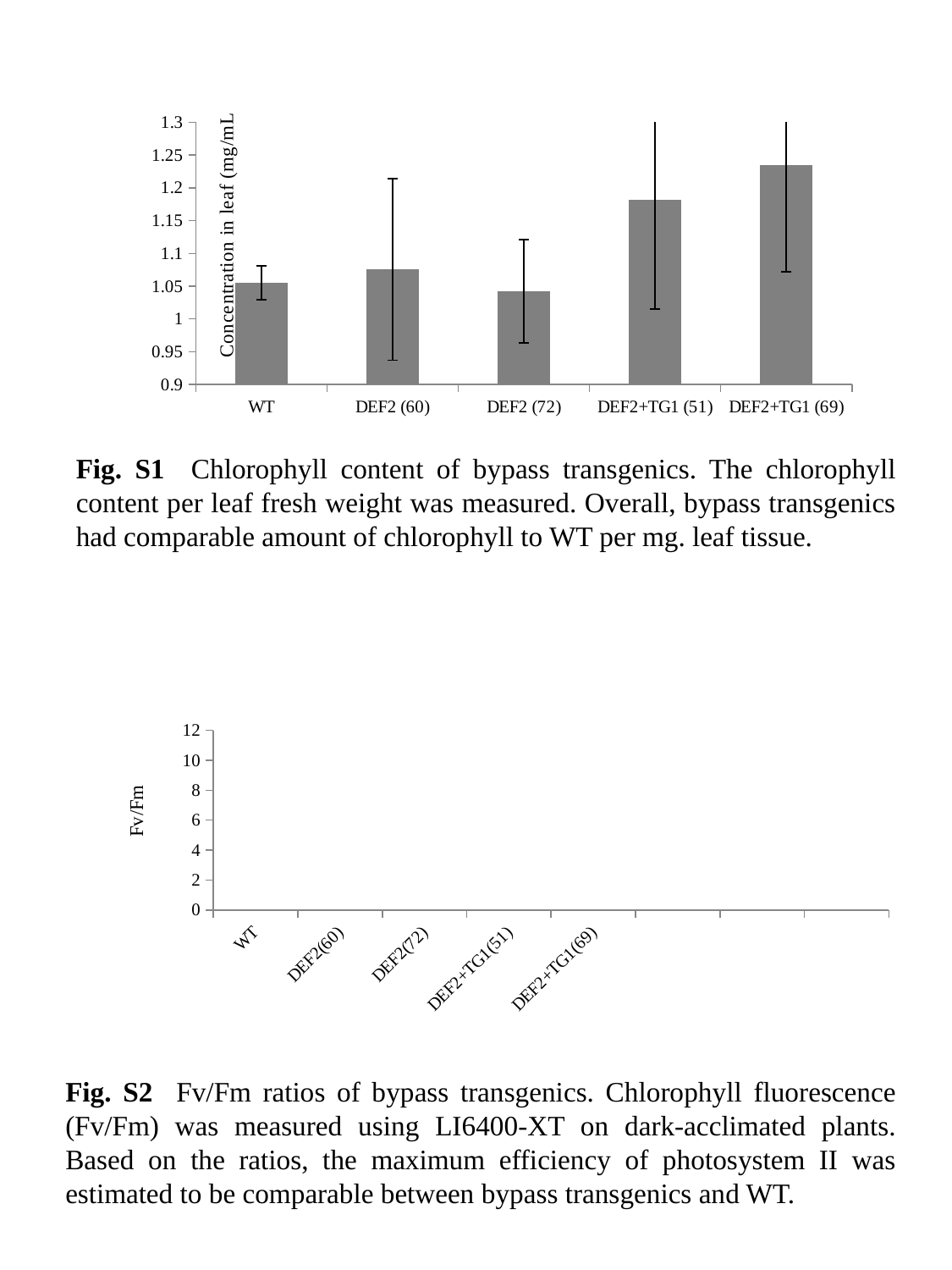

### Chart
| Category | |
|---|---|
| WT | 1.0550280138542674 |
| DEF2 (60) | 1.0755225250990146 |
| DEF2 (72) | 1.042406384971267 |
| DEF2+TG1 (51) | 1.1820244844685561 |
| DEF2+TG1 (69) | 1.2342728680288622 |Fig. S1 Chlorophyll content of bypass transgenics. The chlorophyll content per leaf fresh weight was measured. Overall, bypass transgenics had comparable amount of chlorophyll to WT per mg. leaf tissue.
### Chart
| Category | Fv/Fm |
|---|---|
| WT | 0.8495235157266408 |
| DEF2(60) | 0.8507215456030105 |
| DEF2(72) | 0.8524576991167582 |
| DEF2+TG1(51) | 0.8479147921882755 |
| DEF2+TG1(69) | 0.8509010000000001 |Fig. S2 Fv/Fm ratios of bypass transgenics. Chlorophyll fluorescence (Fv/Fm) was measured using LI6400-XT on dark-acclimated plants. Based on the ratios, the maximum efficiency of photosystem II was estimated to be comparable between bypass transgenics and WT.

## Slide 2
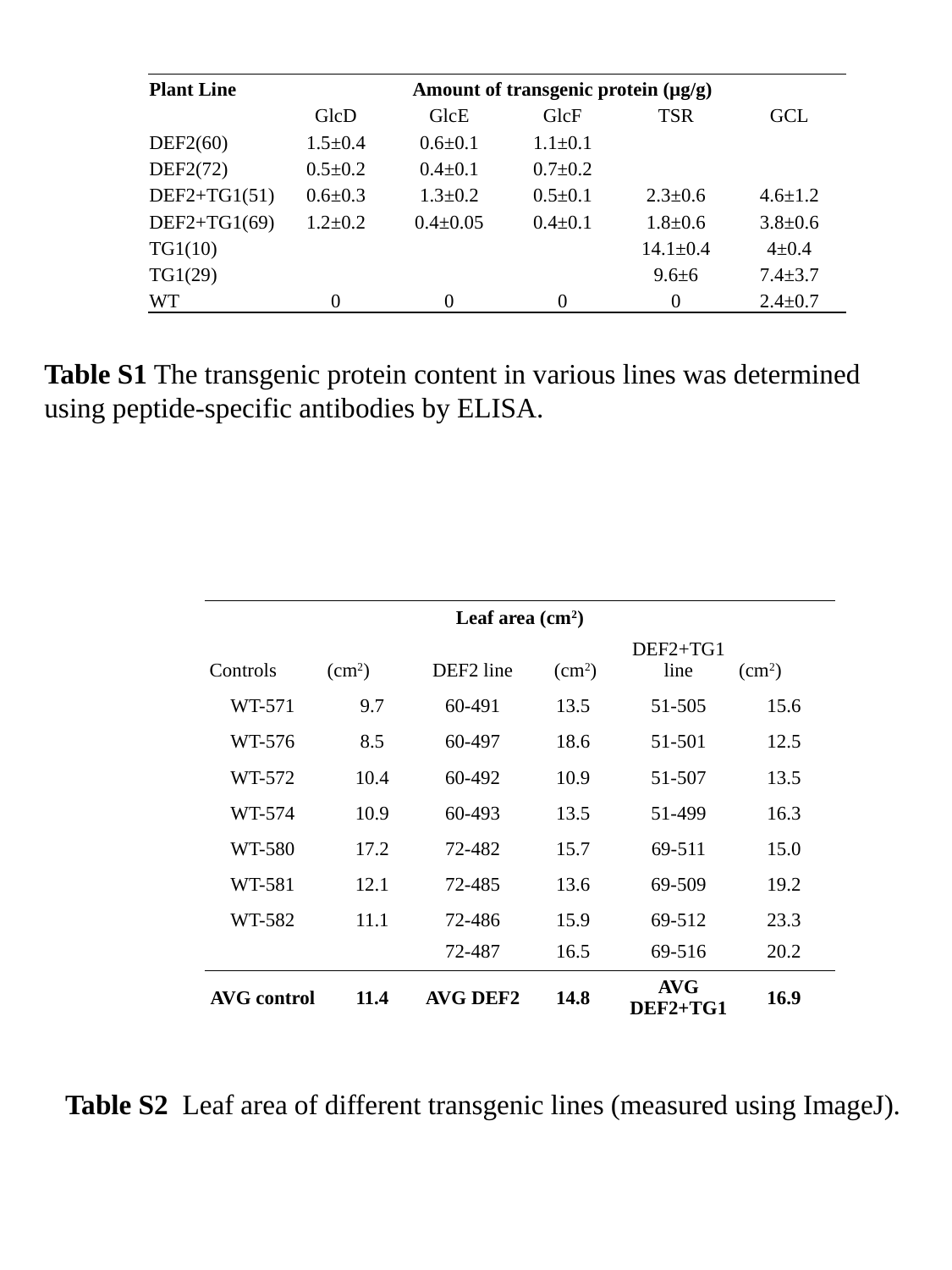

| Plant Line | Amount of transgenic protein (µg/g) | | | | |
| --- | --- | --- | --- | --- | --- |
| | GlcD | GlcE | GlcF | TSR | GCL |
| DEF2(60) | 1.5±0.4 | 0.6±0.1 | 1.1±0.1 | | |
| DEF2(72) | 0.5±0.2 | 0.4±0.1 | 0.7±0.2 | | |
| DEF2+TG1(51) | 0.6±0.3 | 1.3±0.2 | 0.5±0.1 | 2.3±0.6 | 4.6±1.2 |
| DEF2+TG1(69) | 1.2±0.2 | 0.4±0.05 | 0.4±0.1 | 1.8±0.6 | 3.8±0.6 |
| TG1(10) | | | | 14.1±0.4 | 4±0.4 |
| TG1(29) | | | | 9.6±6 | 7.4±3.7 |
| WT | 0 | 0 | 0 | 0 | 2.4±0.7 |
Table S1 The transgenic protein content in various lines was determined using peptide-specific antibodies by ELISA.
| | | | | | |
| --- | --- | --- | --- | --- | --- |
| Leaf area (cm2) | | | | | |
| Controls | (cm2) | DEF2 line | (cm2) | DEF2+TG1 line | (cm2) |
| WT-571 | 9.7 | 60-491 | 13.5 | 51-505 | 15.6 |
| WT-576 | 8.5 | 60-497 | 18.6 | 51-501 | 12.5 |
| WT-572 | 10.4 | 60-492 | 10.9 | 51-507 | 13.5 |
| WT-574 | 10.9 | 60-493 | 13.5 | 51-499 | 16.3 |
| WT-580 | 17.2 | 72-482 | 15.7 | 69-511 | 15.0 |
| WT-581 | 12.1 | 72-485 | 13.6 | 69-509 | 19.2 |
| WT-582 | 11.1 | 72-486 | 15.9 | 69-512 | 23.3 |
| | | 72-487 | 16.5 | 69-516 | 20.2 |
| AVG control | 11.4 | AVG DEF2 | 14.8 | AVG DEF2+TG1 | 16.9 |
| | | | | | |
Table S2 Leaf area of different transgenic lines (measured using ImageJ).

## Slide 3
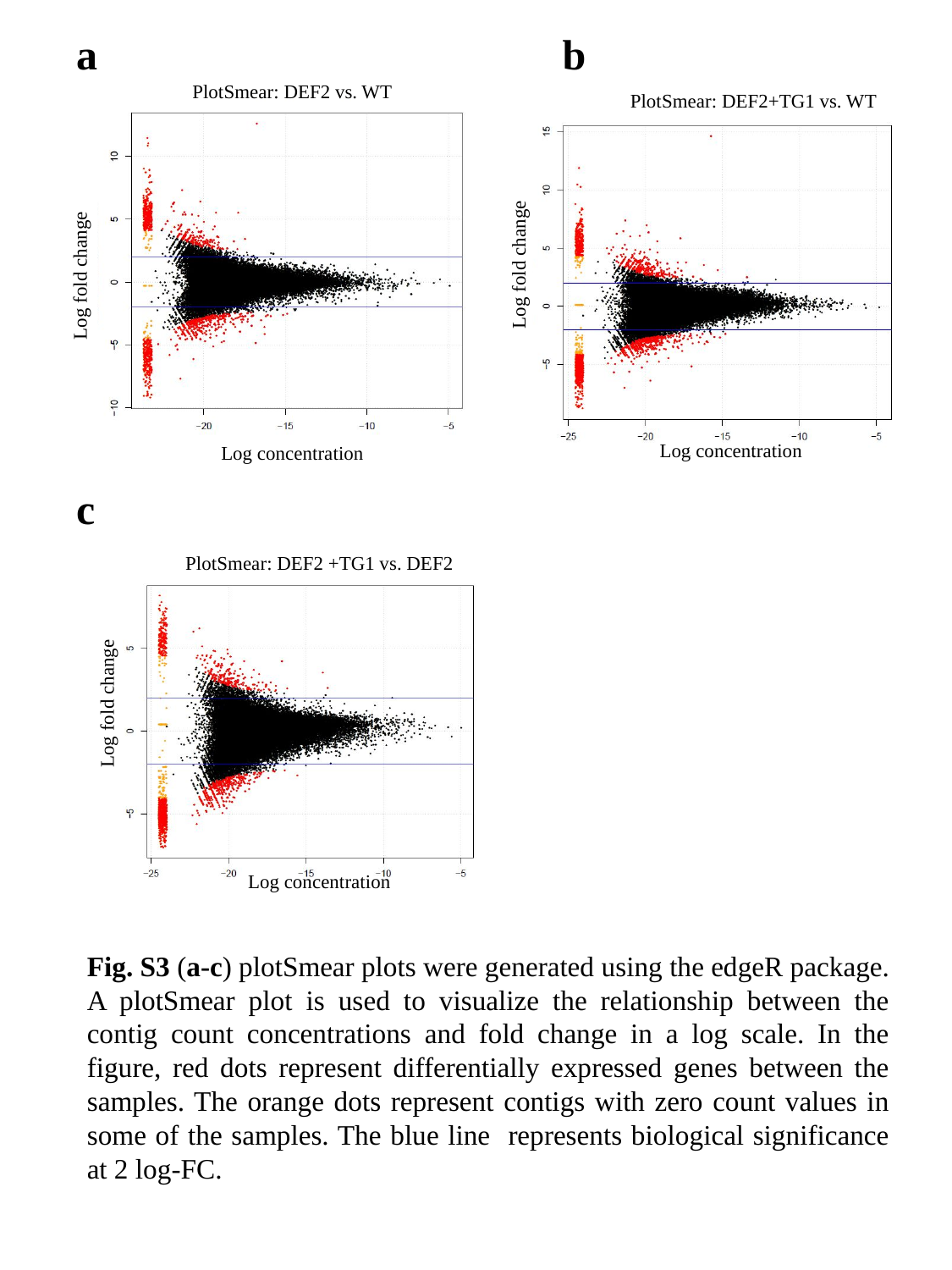

a
b
PlotSmear: DEF2 vs. WT
Log fold change
Log concentration
PlotSmear: DEF2+TG1 vs. WT
Log fold change
Log concentration
c
PlotSmear: DEF2 +TG1 vs. DEF2
Log fold change
Log concentration
Fig. S3 (a-c) plotSmear plots were generated using the edgeR package. A plotSmear plot is used to visualize the relationship between the contig count concentrations and fold change in a log scale. In the figure, red dots represent differentially expressed genes between the samples. The orange dots represent contigs with zero count values in some of the samples. The blue line represents biological significance at 2 log-FC.

## Slide 4
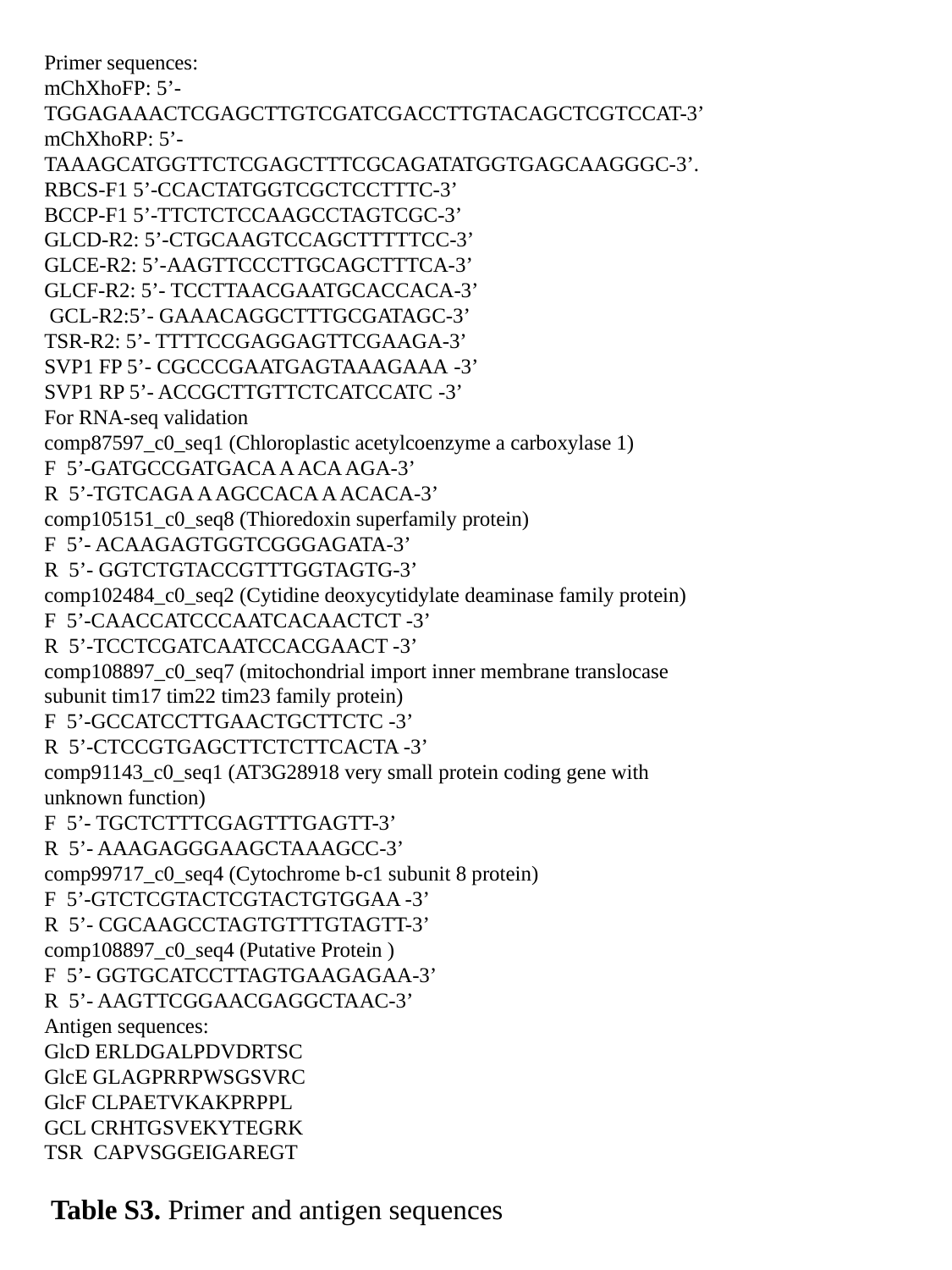

Primer sequences:
mChXhoFP: 5’-TGGAGAAACTCGAGCTTGTCGATCGACCTTGTACAGCTCGTCCAT-3’
mChXhoRP: 5’-TAAAGCATGGTTCTCGAGCTTTCGCAGATATGGTGAGCAAGGGC-3’.
RBCS-F1 5’-CCACTATGGTCGCTCCTTTC-3’
BCCP-F1 5’-TTCTCTCCAAGCCTAGTCGC-3’
GLCD-R2: 5’-CTGCAAGTCCAGCTTTTTCC-3’
GLCE-R2: 5’-AAGTTCCCTTGCAGCTTTCA-3’
GLCF-R2: 5’- TCCTTAACGAATGCACCACA-3’
 GCL-R2:5’- GAAACAGGCTTTGCGATAGC-3’
TSR-R2: 5’- TTTTCCGAGGAGTTCGAAGA-3’
SVP1 FP 5’- cgcccgaatgagtaaagaaa -3’
SVP1 RP 5’- accgcttgttctcatccatc -3’
For RNA-seq validation
comp87597_c0_seq1 (Chloroplastic acetylcoenzyme a carboxylase 1)
F 5’-GATGCCGATGACA A ACA AGA-3’
R 5’-TGTCAGA A AGCCACA A ACACA-3’
comp105151_c0_seq8 (Thioredoxin superfamily protein)
F 5’- ACAAGAGTGGTCGGGAGATA-3’
R 5’- GGTCTGTACCGTTTGGTAGTG-3’
comp102484_c0_seq2 (Cytidine deoxycytidylate deaminase family protein)
F 5’-CAACCATCCCAATCACAACTCT -3’
R 5’-TCCTCGATCAATCCACGAACT -3’
comp108897_c0_seq7 (mitochondrial import inner membrane translocase subunit tim17 tim22 tim23 family protein)
F 5’-GCCATCCTTGAACTGCTTCTC -3’
R 5’-CTCCGTGAGCTTCTCTTCACTA -3’
comp91143_c0_seq1 (AT3G28918 very small protein coding gene with unknown function)
F 5’- TGCTCTTTCGAGTTTGAGTT-3’
R 5’- AAAGAGGGAAGCTAAAGCC-3’
comp99717_c0_seq4 (Cytochrome b-c1 subunit 8 protein)
F 5’-GTCTCGTACTCGTACTGTGGAA -3’
R 5’- CGCAAGCCTAGTGTTTGTAGTT-3’
comp108897_c0_seq4 (Putative Protein )
F 5’- GGTGCATCCTTAGTGAAGAGAA-3’
R 5’- AAGTTCGGAACGAGGCTAAC-3’
Antigen sequences:
GlcD ERLDGALPDVDRTSC
GlcE GLAGPRRPWSGSVRC
GlcF CLPAETVKAKPRPPL
GCL CRHTGSVEKYTEGRK
TSR CAPVSGGEIGAREGT
Table S3. Primer and antigen sequences
